# Mapping the evidence and research gaps on preimplantation genetic testing for aneuploidy in recurrent implantation failure: a scoping review

**DOI:** 10.3389/fendo.2026.1872860

**Published:** 2026-07-27

**Authors:** Ana-Maria Dabuleanu (Cretu), Ovidiu-Dumitru Ilie, Radu Maftei, Mara Doroftei, Bogdan Doroftei

**Affiliations:** 1 Department of Mother and Child, Faculty of Medicine, “Grigore T. Popa” University of Medicine and Pharmacy, Iasi, Romania; 2 “Cuza Voda” Clinical Hospital of Obstetrics and Gynecology, Iasi, Romania; 3 Origyn Fertility Center, Iasi, Romania; 4 Faculty of Medicine, “Grigore T. Popa” University of Medicine and Pharmacy, Iasi, Romania

**Keywords:** clinical pregnancy rate, embryo ploidy, embryo selection, endometrial receptivity, euploid embryo transfer, implantation rate, *in vitro* fertilization, live birth rate

## Abstract

**Background:**

Recurrent implantation failure (RIF) represents a complex and heterogeneous condition in assisted reproductive technology (ART), characterized by unclear etiology and limited evidence-based management strategies. Preimplantation genetic testing for aneuploidy (PGT-A) has been proposed to improve reproductive outcomes through selection of euploid embryos; however, its clinical utility in RIF remains controversial.

**Methods:**

A scoping review was conducted to systematically map the available evidence regarding the role of PGT-A in RIF. A comprehensive literature search identified 18 studies evaluating reproductive outcomes in RIF patients undergoing PGT-A compared with conventional *in vitro* fertilization/intracytoplasmic sperm injection (IVF/ICSI). Data were extracted and narratively synthesized across key outcomes, including implantation rate (IR), clinical pregnancy rate (CPR), live birth rate (LBR), and miscarriage rate (MR). Methodological quality was assessed using the Newcastle-Ottawa Scale (NOS), and sources of heterogeneity, including variability in RIF definitions, PGT-A methodologies, and patient subgroups, were also explored.

**Results:**

Several studies reported improved outcomes at the level of individual embryo transfer (ET), including higher IR, CPR, and LBR per transfer following PGT-A. However, these findings were not consistently observed when outcomes were assessed at the patient level. In particular, cumulative live birth rate (CLBR) was generally comparable between PGT-A and non-PGT-A groups, especially in unexplained - uRIF or idiopathic - iRIF populations. Similarly, MR was largely unchanged across studies despite improved embryo selection. Subgroup analyses suggested that PGT-A may provide greater benefit in patients with a higher likelihood of embryo-related implantation failure (IF), such as those of advanced maternal age (AMA), whereas limited benefit was observed in uRIF/iRIF cohorts. Overall, substantial heterogeneity was identified regarding RIF definitions, laboratory methodologies, and clinical protocols.

**Conclusions:**

The available evidence suggests that PGT-A may improve treatment efficiency by increasing the probability of success per ET in selected RIF populations. Nevertheless, it does not consistently improve CLBR or reduce miscarriage risk, underscoring the multifactorial nature of IF. Consequently, PGT-A should be considered within an individualized treatment strategy rather than as a universal intervention for all RIF patients. Further prospective studies using standardized definitions and protocols are required to better define its clinical role.

## 1 Introduction

RIF remains one of the most challenging and incompletely understood conditions in ART. Despite substantial advances in IVF, a proportion of patients repeatedly fails to achieve implantation even after the transfer of morphologically high-quality embryos. One of the major limitations in this field is the absence of a universally accepted definition of RIF, with diagnostic criteria varying considerably across studies according to the number of failed ET, embryo quality, and developmental stage (1, 2). This heterogeneity reflects the complex and multifactorial nature of IF, which involves not only embryo competence but also endometrial receptivity (ER), uterine factors, immunological mechanisms, and systemic maternal conditions (3, 4).

Embryo aneuploidy has been consistently identified as an important contributor to IF and early pregnancy loss, particularly in women of AMA (5, 6). This biological rationale led to the development of PGT-A, which aims to improve reproductive outcomes through the selection of euploid embryos prior to transfer. Technological advances, particularly the transition from array comparative genomic hybridization (aCGH) to next-generation sequencing (NGS), have improved the resolution of chromosomal assessment and enabled the detection of embryonic mosaicism (7, 8).

Despite this strong biological rationale, the clinical effectiveness of PGT-A remains controversial. Recent meta-analyses suggest that PGT-A may improve reproductive outcomes at the level of individual ET, including IR, CPR, and LBR, particularly in selected patient populations (9, 10). However, these benefits are less consistent when outcomes are evaluated at the patient level, especially regarding CLBR, for which no clear advantage has been demonstrated in unselected IVF populations (11). Moreover, concerns persist regarding embryo attrition, the clinical significance of mosaic embryos, false-positive aneuploidy findings, and the potential impact of trophectoderm (TE) biopsy on embryo viability (7, 12).

These uncertainties are reflected in current clinical guidelines. The Practice Committee of the American Society for Reproductive Medicine (ASRM, 2024) (12) concluded that the routine use of PGT-A in IVF remains controversial, emphasizing that the available evidence is heterogeneous and insufficient to support universal application. Importantly, RIF itself is unlikely to represent a single biological entity. Instead, emerging evidence suggests that IF may arise predominantly from embryo-related mechanisms, endometrial dysfunction, or a combination of both, potentially explaining the heterogeneous clinical effectiveness of PGT-A across patient populations. Another major challenge in interpreting the available evidence is the substantial methodological heterogeneity across studies. Variability in RIF definitions, patient populations, embryo stage at transfer, PGT-A platforms, mosaicism thresholds, and embryo classification criteria significantly limits comparability and contributes to conflicting findings (2, 3). Furthermore, most systematic reviews and meta-analyses have evaluated PGT-A in heterogeneous IVF populations rather than focusing specifically on RIF, thereby limiting the applicability of their conclusions to this distinct clinical condition.

Therefore, the aim of the present scoping review is to systematically map and synthesize the current evidence regarding the use of PGT-A in patients with RIF. Specifically, this review seeks to evaluate reproductive outcomes associated with PGT-A, including IR, CPR, LBR, and MR, while exploring how embryo ploidy status influences implantation success in this population. In addition, we propose a conceptual framework distinguishing embryo-, endometrium-driven, and mixed forms of RIF, with the goal of providing a biologically grounded perspective on patient selection and the clinical role of PGT-A in this heterogeneous condition.

## 2 Materials and methods

### 2.1 Methodology

The present scoping review was designed in accordance with the Preferred Reporting Items for Systematic Reviews and Meta-Analyses extension for Scoping Reviews (PRISMA-ScR) 2018 guidelines (13, 14), ensuring a structured and transparent approach to evidence mapping.

### 2.2 Ethical statement

No Institutional Review Board (IRB) approval or informed consent was required, as this study is based exclusively on data previously published in peer-reviewed journals.

### 2.3 Objectives

The present scoping review was designed to systematically map and synthesize the available evidence regarding the role of PGT-A in patients with RIF. Given the heterogeneity of the literature and the absence of consensus regarding the clinical utility of PGT-A in this setting, the review aimed to explore patterns of evidence, summarize reported reproductive outcomes, and identify knowledge gaps and areas of ongoing controversy.

### 2.4 Research questions

#### 2.4.1 Primary question

What evidence is currently available regarding the use of PGT-A in patients with RIF?

#### 2.4.2 Secondary questions

What reproductive outcomes have been reported in RIF patients undergoing PGT-A?

What evidence exists regarding the effect of PGT-A on miscarriage outcomes in RIF?

How does embryo ploidy status influence implantation success in patients with RIF?

### 2.5 Sources inquiry

A comprehensive literature search was performed across four major electronic databases: PubMed-MEDLINE - United States National Library of Medicine (NLM, 1996), Web of Science™ (WOS) (Clarivate Analytics, 1997), Scopus (Elsevier, 2004), and Excerpta Medica dataBASE (EMBASE) (Elsevier, 1947). These sources were selected due to their complementary indexing systems and broad coverage of biomedical research, allowing for a more inclusive retrieval of relevant studies (15–18).

The search covered the period from April 1st, 2016, to April 1st, 2026, focusing on recent developments and clinically applicable evidence in the field. This timeframe was selected because it encompasses the transition toward contemporary PGT-A practices, including blastocyst-stage TE biopsy and NGS-based chromosomal screening, which have become the predominant clinical approaches. Earlier studies frequently relied on cleavage-stage biopsy and fluorescence *in situ* hybridization (FISH), methodologies that are no longer routinely used and may not accurately reflect current clinical practice.

The search strategy relied primarily on free-text keywords, supplemented where applicable by controlled vocabulary. The core terms included “Recurrent Implantation Failure”, “RIF”, “Repeated Implantation Failure”, “Preimplantation Genetic Testing”, “Pre-implantation Genetic Testing”, and “Aneuploidy”.

Among these, only “Aneuploidy” is formally indexed as a MeSH term; [Aneuploidy]: MeSH Unique ID: D000782, Tree Number(s): C23.550.210.050, G05.365.590.175.050, G05.700.131. The remaining terms are not standardized descriptors within MeSH and were therefore applied as free-text keywords, along with their relevant synonyms, in order to enhance the sensitivity of the search strategy.

Broader reproductive terms were deliberately excluded from the primary search framework. Their inclusion was considered likely to substantially broaden the scope of the search and reduce its specificity relative to the targeted research questions.

### 2.6 String strategy

Boolean operators, including “AND” and “OR” were used to construct the primary search (Search #1) as a single query. Additional combinations and refinements were subsequently applied to develop a secondary search strategy (Search #2), aimed at optimizing the balance between sensitivity and specificity. The complete search strings are provided in **Supplementary File 1**.

### 2.7 Selection of studies

All retrieved references were imported into Mendeley - Reference Management Software (v. 1.19.8) (Elsevier, 2013), for organization and deduplication. This software has demonstrated a high level of accuracy in identifying duplicate records, with performance comparable to other established platforms such as Ovid/Rayyan and Covidence (19).

Automatic duplicate detection was followed by manual verification to ensure consistency across records. Titles and abstracts were independently screened by all contributors, followed by full-text evaluation of studies meeting the predefined eligibility criteria. Full-text assessment was conducted by A.-M.D. (C.)., O.-D.I., and M.D.

Any discrepancies encountered during the selection process were resolved through discussion until consensus was reached.

### 2.8 Extraction of data

Data extraction was carried out by A.-M.D. (C.)., O.-D.I., R.M., and B.D. using a standardized table developed in Microsoft Excel 2010 (Microsoft Corporation, Redmond, WA, USA). This tool also facilitated data organization, categorization, and synthesis.

The extracted information included general study characteristics such as author, year of publication, country, study design, and sample size. In addition, specific variables relevant to the research objectives were recorded, including the definition of RIF applied in each study, the type of PGT-A methodology used, and the developmental stage of the embryos analyzed.

Clinical outcomes were also systematically documented, including IR, CPR, LBR, and MR, in order to support a comprehensive overview of the reported findings.

### 2.9 Criteria of inclusion and exclusion

Studies were eligible for inclusion if they presented original research data, involved human participants, and specifically assessed the use of PGT-A in patients diagnosed with RIF. Only articles published in English and indexed in peer-reviewed journals were considered, regardless of study design.

Studies were excluded if they represented secondary literature, particularly reviews, meta-analyses, editorials, or commentaries, were based on animal or *in vitro* models, or did not include a clearly defined RIF population. Additionally, studies lacking relevant clinical outcome data were excluded from the analysis.

### 2.10 Methodological quality assessment

The quality of the included observational studies was assessed by A.-M.D. (C.)., O.-D.I., and B.D. using the NOS for cohort studies (20). The NOS evaluates studies across three domains: Selection (maximum four stars), Comparability (maximum two stars), and Outcome (maximum three stars), yielding a maximum score of nine stars.

Studies were independently assessed according to the NOS coding manual. The following thresholds were adopted for interpretation: studies scoring 7–9 stars were considered of high methodological quality, those scoring 5–6 stars were considered of moderate quality, and studies scoring ≤4 stars were classified as low quality. Disagreements regarding study appraisal were resolved through discussion and consensus. The NOS was used to assess methodological quality in observational studies; however, it should not be interpreted as a formal measure of evidence certainty. Given the scoping nature of the present review and the predominance of retrospective observational studies, the quality assessment was intended to contextualize the findings rather than to support quantitative comparisons or evidence grading.

## 3 Results

### 3.1 Number of results and final inclusion

**Figure 1:**
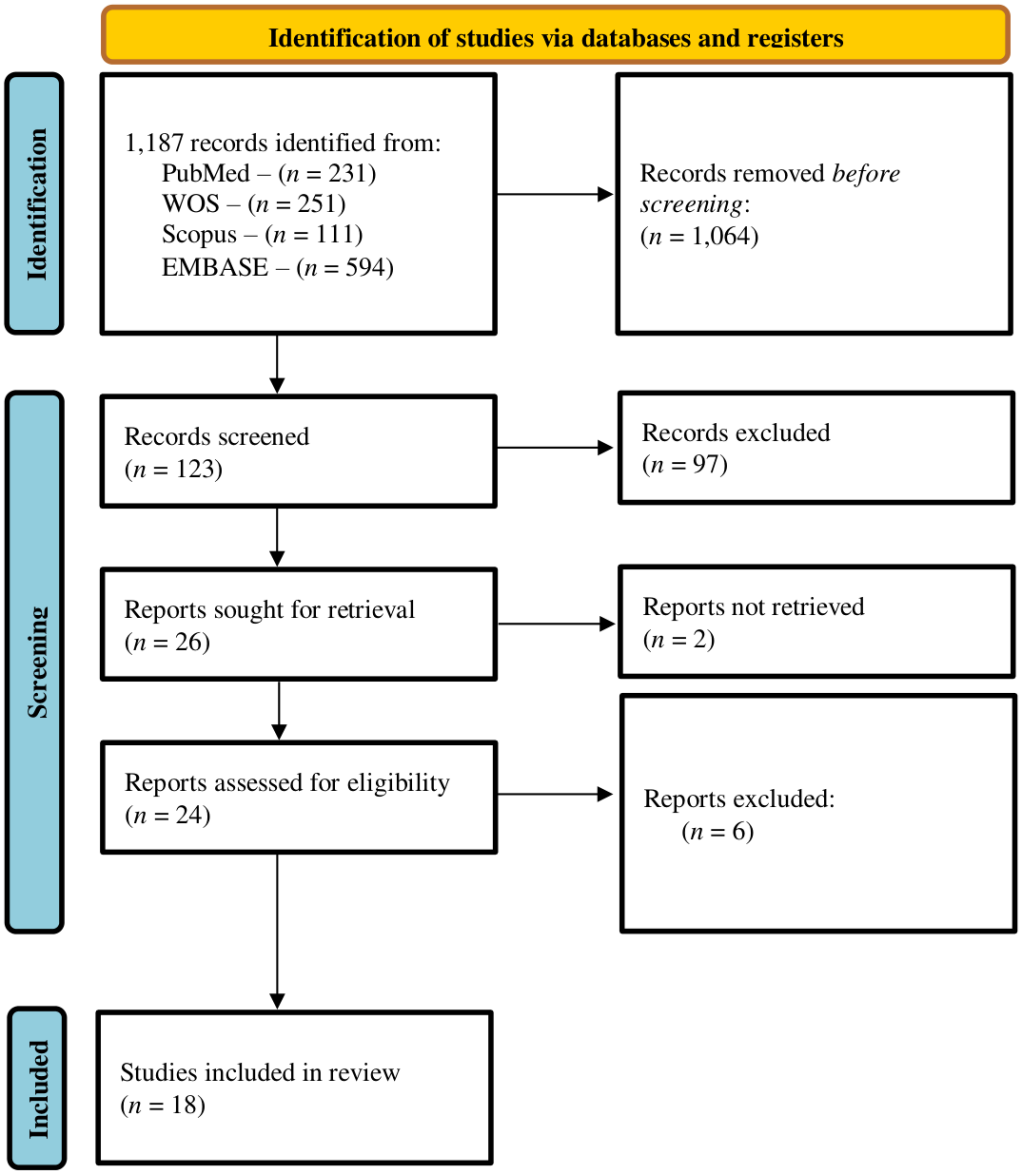
PRISMA-ScR diagram.

A total of 1,187 records were identified through database searching, including 231 (19.46%) were retrieved from PubMed-MEDLINE, 251 (21.14%) from WOS, 111 (9.35%) from Scopus, and 594 (50.04%) from EMBASE. After removing 1,064 duplicate records prior to screening, 123 records remained for title and abstract screening. Following this step, 97 records were excluded based on title and abstract evaluation, and 26 reports were sought for retrieval. Of these, 2 reports were not retrieved, leaving 24 full-text articles assessed for eligibility. Subsequently, 6 reports were excluded after full-text review because of lack of relevance to the research question, inappropriate study design, absence of a clearly defined RIF population, or insufficient reporting of clinical outcomes. Ultimately, 18 studies met the eligibility criteria and were included in the present scoping review (**Figure 1**, **Table 1**).

**Table 1:** Characteristics of included studies.

Author (year)	Country	Study design	Population	Sample size	*RIF definition	*PGT-A method	Comparator	*Main outcomes
(Lee et al., 2019) (21)	Taiwan	Retrospective study	High-risk IVF patients: AMA, RIF, RM, OD controls	n = 296 couples; *n* = 87 AMA *n* = 82 RIF *n* = 82 RM *n* = 45 OD *n* = 61 AMA non-PGT-A controls	≥3 failed IVF cycles with transfer of good-quality embryos	Blastocyst-stage biopsy + aCGH	Non-PGT-A comparator only for AMA; no explicit RIF non-PGT-A comparator for the RIF subgroup	IR, CPR, LBR, MR
(Sato et al., 2019) (22)	Japan	Multicenter prospective pilot study	Patients with RPL and RIF undergoing IVF-ET	*n* = 171 patients; RPL group; *n* = 41 with PGT-A *n* = 38 without PGT-A RIF group; *n* = 42 with PGT-A *n* = 50 without PGT-A	No pregnancy after ≥3 good-quality blastocysts transfer	TE biopsy + aCGH + WGA	PGT-A vs. non-PGT-A	CPR, LBR, MR
(Cozzolino et al., 2020) (23)	Multicenter Europe	Retrospective multicenter cohort study	Moderate and severe RIF patients	*n* = 2,598 patients; *n* = 2,110 moderate RIF *n* = 488 severe RIF	≥3 embryos transferred in different SET without implantation (moderate RIF) ≥5 or more embryos in at least 3 SET (severe RIF)	Blastocyst-stage biopsy + aCGH or NGS	PGT-A, ERA, PGT-A + ERA vs. no testing	IR
(Tong et al., 2021) (24)	China	Retrospective study	RIF patients undergoing PGT-A, stratified by female age (<38 vs. ≥38 years)	*n* = 265 couples; 346 OR cycles; 250 PGT-A cycles *n* = 184 couples; 221 OR cycles, 180 NGS cycles *n* = 81 couples, 125 OR cycles, 70 NGS cycles	Failure after two consecutive IVF/ICSI or FET cycles, with ≥4 cleavage-stage embryos or ≥2 blastocysts transferred of good quality	TE biopsy + NGS	No non-PGT-A control; comparison was between younger and older PGT-A-treated RIF groups	IR, CPR, MR
(Fodina et al., 2021) (25)	Latvia	Retrospective study	Patients with RIF undergoing ICSI cycles	*n* = 253 cycles *n* = 72 cycles; group I – FET without testing *n* = 87; group II – FET with PGT-A *n* = 72; group III – FET with PGT-A and ERA *n* = 22; group IV – FET with ERA	Failure to achieve a clinical pregnancy after transfer of ≥3 good-quality embryos in different single fresh or frozen ET	TE biopsy + WGA + aCGH or NGS	no testing vs. PGT-A vs. PGT-A + ERA vs. ERA	CPR, MR
(Pantou et al., 2022) (26)	Greece	Retrospective cohort study	High-risk patients: AMA, RM, RIF	*n* = 455 patients; *n* = 121 AMA PGT-A *n* = 25 RM PGT-A *n* = 30 RIF PGT-A *n* = 197 AMA control *n* = 40 RM control *n* = 42 RIF control	≥4 good-quality embryos in ≥3 IVF cycles (maternal age < 40 years)	Blastocyst-stage biopsy + aCGH	PGT-A groups vs. similarly subgrouped non-PGT-A controls	IR, LBR, MR
(Amin et al., 2022) (27)	India	Retrospective observational study	RIF patients undergoing ERA-guided personalized ET; some also underwent PGT-A	*n* = 291 patients; *n* = 72 Control group *n* = 219 ERA group	>2 failed fresh/frozen ET with two morphologically normal or PGT-A-normal embryos; self and donor cycles included	TE biopsy + NGS	Group I without ERA vs. Group II ERA; within ERA group, PGT-A-screened vs. non-PGT-A-screened patients	IR, CPR
(Wang et al., 2023) (28)	China	Retrospective cohort study	Women <40 years with uRIF with PGT-A, uRIF without PGT-A, and non-RIF with PGT-A	*n* = 154 patients; *n* = 29 uRIF + PGT-A *n* = 87 uRIF + non-PGT-A *n* = 38 non-uRIF + PGT-A	≥3 consecutive transfers of good-quality embryos	Blastocyst-stage PGT-A + NGS	uRIF + PGT-A vs. uRIF + non-PGT-A; additional non-uRIF + PGT-A group	CPR, LBR, CLBR, MR
(Shi et al., 2023) (29)	China	Retrospective cohort study	iRPL and iRIF couples	*n* = 311 couples; *n* = 212 iRPL *n* = 66 iRIF	Absence of implantation after two consecutive IVF/ICSI or FET cycles, with a cumulative transfer of ≥4 cleavage-stage embryos or ≥2 blastocysts, all of good quality	TE biopsy + NGS	PGT-A vs. conventional IVF/ICSI	IR, CPR, LBR, MR
(Du et al., 2023) (30)	China	Retrospective cohort study	RIF patients <38 years undergoing first FET after RIF diagnosis	*n* = 178 patients; *n* = 59 PGT-A *n* = 119 Control	Failure to achieve pregnancy after ≥3 fresh or frozen cycles, or after cumulative transfer of ≥4 high-quality cleavage-stage embryos or ≥2 good-quality blastocysts	TE biopsy + WGA + NGS	PGT-A vs. non-PGT-A	CPR, MR
(Gu et al., 2023) (31)	China	Retrospective case-control study	Women with RIF undergoing IVF	*n* = 466 patients; *n* = 209 RIF-PGT *n* = 257 RIF-non-PGT	Failure after ≥3 ET cycles or ≥4 high-grade embryos transferred	Blastocyst-stage biopsy + SNP-array or NGS	PGT-A vs. non-PGT-A	CPR, LBR, MR
(Kato et al., 2023a) (32)	Japan	Retrospective cohort study	Patients with RIF and RPL	*n* = 7,668 records reviewed; *n* = 579 PGT-A matched 1:1 within a control pool of 7,089 non-PGT-A patients	History of ≥3 IF after IVF-ET treatment	Blastocyst-stage biopsy + WGA + aCGH or NGS	PGT-A vs. non-PGT-A control	IR, CPR, LBR, MR
(Kato et al., 2023b) (33)	Japan	Retrospective cohort study	Women aged 35–42 with RPL or RIF in minimal stimulation IVF	*n* = 2,588 patients; *n* = 2556 Control *n* = 18 RIF-PGT-A *n* = 14 RPL-PGT-A	Failure after ≥3 failed IVF-ET cycles	Blastocyst-stage biopsy + WGA + aCGH	PGT-A vs. non-PGT-A	IR, CPR, LBR, MR
(Kozyra et al., 2024) (34)	Ukraine/Italy	Prospective cohort study	Infertile women with RIF undergoing IVF	*n* = 93 infertile women	>2 IF	Blastocyst-stage biopsy + NGS	PGT-A + personalized ET vs. PGT-A	LBR
(Kim et al., 2024) (35)	Republic of Korea	Retrospective cohort study	High-risk patients: AMA, RIF, RPL, SMF infertility	*n* = 1,368 patients/cycles *n* = 233 AMA, *n* = 130 RIF, *n* = 80 RPL, and *n* = 77 SMF - PGT-A *n* = 289 AMA, *n* = 266 RIF, *n* = 132 RPL, *n* = 161 SMF - without PGT-A	Absence of a gestational sac on ultrasound following transfer in ≥3 fresh or frozen cycles	Blastocyst-stage biopsy + aCGH or NGS	PGT-A vs. non-PGT-A	IR, CPR, OPR/LBR, MR
(Gajjar et al., 2024) (36)	India, USA	Retrospective cohort study	RIF patients undergoing OpERA-guided personalized ET	*n* = 158 patients *n* = 38; ≤1 prior failed ET *n* = 67; 2 prior failed ET *n* = 53; ≥3 prior failed ET	History of failed ET; subgroup with ≥3 failed transfers	Blastocyst-stage PGT-A + NGS	OpERA + PGT-A vs. OpERA without PGT-A	IR, CPR, MR
(Mei et al., 2024) (37)	China	Retrospective study	Patients with uRPL or uRIF in ICSI cycles	*n* = 97 patients; *n* = 49 RPL PGT *n* = 23 RPL non-PGT *n* = 10 RIF PGT *n* = 15 non-PGT	≥3 failed transfers of good-quality embryos in women aged between 20–44 years	TE biopsy + NGS	PGT-A vs. non-PGT-A	CPR, MR
(Liu et al., 2024) (38)	China	Retrospective cohort study	Couples with uRIF	*n* = 705 couples; *n* = 476 PGT-A *n* = 229 IVF/ICSI	Failure after ≥3 ET or ≥3 blastocysts/≥4–6 good-quality cleavage embryos	TE biopsy + NGS	PGT-A vs. IVF/ICSI	LBR, MR

*Definitions of RIF, PGT-A methodologies, and reported clinical outcomes varied considerably across studies, contributing to significant heterogeneity.

IVF, *in vitro* fertilization; AMA, advanced maternal age; RIF, recurrent implantation failure; RM, recurrent miscarriage; OD, oocyte donor; aCGH, array comparative genomic hybridization; IR, implantation rate; CPR, clinical pregnancy rate; LBR, live birth rate; MR, miscarriage rate; RPL, recurrent pregnancy loss; ET, embryo transfer; PGT-A, preimplantation genetic testing for aneuploidy; TE, trophectoderm; WGA, whole genome amplification; SET, single ET; NGS, next-generation sequencing; OR, oocyte retrieved; FET, frozen ET; ICSI, intracytoplasmic sperm injection; ERA, endometrial receptivity assay; uRIF, unexplained RIF; CLBR, cumulative LBR; iRPL, idiopathic RPL; iRIF, idiopathic RIF; SNP, single nucleotide polymorphism; IF, implantation failure; SMF, severe male factor; OpERA, Optimal Timing for Endometrial Receptivity Analysis; uRPL, unexplained RPL.

### 3.2 Methodological quality assessment and narrative synthesis of the evidence

**Figure 2:**
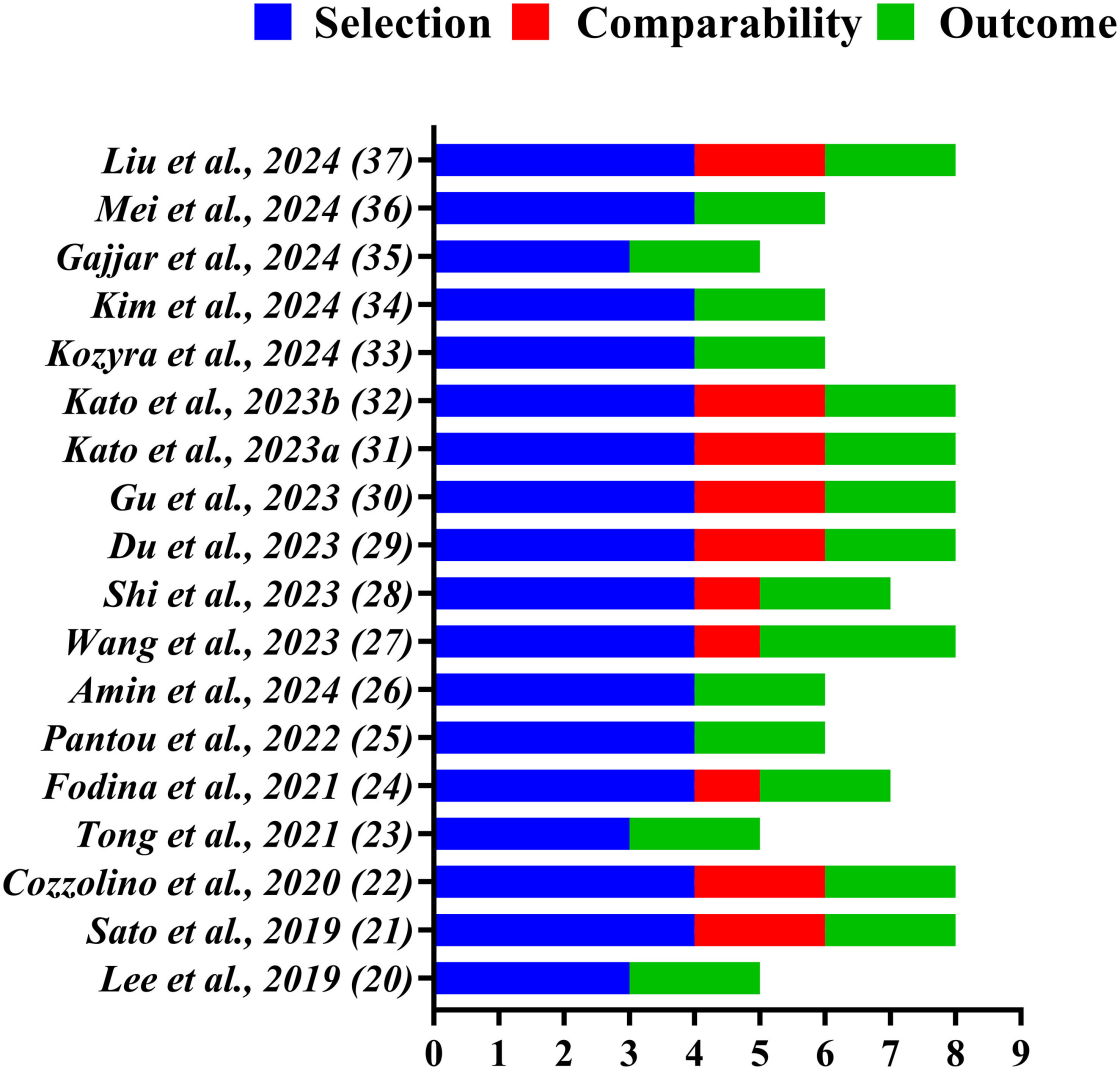
NOS assessment of the included studies. Bar plots illustrate the scores obtained by each study in the three NOS domains: Selection (blue; maximum 4 stars), Comparability (red; maximum 2 stars), and Outcome (green; maximum 3 stars). Total NOS scores ranged from 5 to 8 stars, reflecting overall moderate-to-high methodological quality across the included studies.

Among the 18 included studies, NOS scores ranged from 5 to 8 stars, indicating an overall moderate-to-high methodological quality of the available evidence (**Table 2**, **Figure 2**). Eight studies were classified as high quality (NOS score = 8), two studies achieved a score of 7, five studies were rated as moderate quality with 6 stars, and three studies received 5 stars. No study was categorized as low quality.

**Table 2:** Methodological quality assessment of the included studies according to the NOS scale.

Study	Representativeness of the exposed cohort	Selection of the non-exposed cohort	Ascertainment of exposure	Demonstration that outcome of interest was not present at start of study	Comparability of cohorts on the basis of the design or analysis	Assessment of outcome	Was follow-up long enough for outcomes to occur	Adequacy of follow up of cohorts	Overall score
(Lee et al., 2019) (21)	*	–	*	*	–	*	*	–	5
(Sato et al., 2019) (22)	*	*	*	*	**	*	*	–	8
(Cozzolino et al., 2020) (23)	*	*	*	*	**	*	*	–	8
(Tong et al., 2021) (24)	*	–	*	*	–	*	*	–	5
(Fodina et al., 2021) (25)	*	*	*	*	*	*	*	–	7
(Pantou et al., 2022) (26)	*	*	*	*	–	*	*	–	6
(Amin et al., 2022) (27)	*	*	*	*	–	*	*	–	6
(Wang et al., 2023) (28)	*	*	*	*	*	*	*	*	8
(Shi et al., 2023) (29)	*	*	*	*	*	*	*	–	7
(Du et al., 2023) (30)	*	*	*	*	**	*	*	–	8
(Gu et al., 2023) (31)	*	*	*	*	**	*	*	–	8
(Kato et al., 2023a) (32)	*	*	*	*	**	*	*	–	8
(Kato et al., 2023b) (33)	*	*	*	*	**	*	*	–	8
(Kozyra et al., 2024) (34)	*	*	*	*	–	*	*	–	6
(Kim et al., 2024) (35)	*	*	*	*	–	*	*	–	6
(Gajjar et al., 2024) (36)	*	–	*	*	–	*	*	–	5
(Mei et al., 2024) (37)	*	*	*	*	–	*	*	–	6
(Liu et al., 2024) (38)	*	*	*	*	**	*	*	–	8

Newcastle–Ottawa Scale (NOS) scoring: “—” indicates that no point (no star) was awarded for a given item (0 points), “*” indicates that one point (one star) was awarded (1 point), and “**” indicates that two points (two stars) were awarded (2 points), where applicable according to the NOS scoring criteria.

The most frequent methodological limitations were related to the Comparability and Outcome domains. Several studies did not employ matching strategies or multivariable adjustment for important confounding variables, resulting in lower comparability scores. In addition, many studies did not explicitly report losses to follow-up or provide sufficient information regarding follow-up completeness, precluding the assignment of a star in the adequacy of follow-up domain according to the NOS manual.

Despite the generally favorable NOS scores, caution is warranted when interpreting the available evidence. Most studies were retrospective cohorts conducted in single centers, with relatively small sample sizes and heterogeneous inclusion criteria. Furthermore, the definition of RIF varied considerably across studies, ranging from two to three failed ET cycles or the transfer of a variable number of good-quality embryos. Additional heterogeneity arose from differences in PGT-A platforms (aCGH, SNP array, or NGS), biopsy strategies, ET policies, and the reporting of reproductive outcomes.

Consequently, although several studies suggested improved IR, CP, or LBR per ET following PGT-A, the overall body of evidence should be interpreted with caution. The methodological heterogeneity and observational nature of the available literature limit the strength of causal inferences and preclude definitive conclusions regarding the routine use of PGT-A in patients with RIF.

### 3.3 Implantation rate and clinical pregnancy

Overall, the available evidence indicates that PGT-A is associated with higher IR and CPR in several RIF cohorts, particularly when outcomes are assessed per ET. However, these findings were not consistent across all studies, and substantial heterogeneity in patient selection, RIF definitions, embryo quality, and adjunctive endometrial interventions limits direct comparison and precludes definitive conclusions.

In the prospective study by Sato et al. (22), CPR per ET was significantly higher after PGT-A than in the non-PGT-A group (70.8% vs. 31.7%, *p* = 0.003). Similarly, Pantou et al. (26), reported significantly higher IR in RIF patients undergoing PGT-A compared with RIF controls, with IR increasing from 33.3% to 69.5%, *p* = 0.005), as well as higher live birth per ET (47.8% vs. 19.0%, *p* = 0.015). Larger retrospective cohorts supported this direction of effect. Gu et al. (31), demonstrated significantly higher human chorionic gonadotropin (hCG) positivity and CPR in the RIF-PGT-A group both before and after propensity score matching. After matching, hCG positivity (56.9% vs. 33.9%, *p* = 0.001) and CPR (49.5% vs. 31.2%, *p* = 0.006) remained significantly higher in the PGT-A group. Likewise, Wang et al. (28), reported higher CPR per transfer in RIF + PGT-A compared with RIF + no PGT-A (52.4% vs. 39.0%, *p* = 0.040), whereas Du et al. (30), observed significantly higher CPR among young RIF patients undergoing PGT-A (71.19% vs. 56.30%, *p* = 0.039). Collectively, these findings suggest that euploid embryo selection may increase the probability of achieving clinical pregnancy in a single transfer attempt.

However, the benefit appeared more nuanced when PGT-A was evaluated alongside endometrial interventions. Cozzolino et al. (23), found that PGT-A improved IR in moderate RIF compared with standard IVF (45.9% vs. 35.9%, *p* < 0.001), whereas this effect was not evident in severe RIF and was not enhanced by combining PGT-A with ERA. Fodina et al. (25), similarly reported significantly higher biochemical and CPR rates only in FET + PGT-A group compared with standard FET (*p* = 0.01 and *p* = 0.049, respectively), whereas ERA alone or ERA combined with PGT-A did not demonstrate superior outcomes. In contrast, studies using personalized endometrial approaches reported more encouraging results. Gajjar et al. (36), observed significantly higher IR and CPR when PGT-A was combined with OpERA (IR: 52.6% vs. 27.2%, *p* = 0.0001; CPR: 62.8% vs. 46.9%, *p* = 0.0467). Similarly, Amin et al. (27), reported that RIF patients undergoing both ERA-guided personalized ET and PGT-A achieved higher reproductive outcomes than those without embryo genetic screening. IR ranged from 88% to 100% in the PGT-A groups compared with 57% to 96% in the non-PGT-A groups (*p* = 0.005 and *p* = 0.01) for the receptive and non-receptive ERA subgroups. CPR rates were also numerically higher in patients undergoing PGT-A, reaching 88% and 95% in the receptive and non-receptive subgroups, respectively, compared with 34% and 37% in patients without PGT-A (*p* = 0.008 and *p* = 0.08, respectively). These findings suggest that embryo chromosomal competence and endometrial synchronization may act synergistically in selected RIF populations. Conversely, several studies failed to demonstrate a clear advantage of PGT-A, particularly in iRIF or uRIF. Tong et al. (24), found no significant difference in IR or CPR between younger and older RIF patients after euploid ET (IR: 51.0% vs. 39.1%; CPR: 48.0% vs. 39.1%; both non-significant), suggesting that maternal age may exert a more limited influence once euploid embryos are selected. In iRIF and uRIF, Shi et al. (29), reported comparable IR (50% vs. 46.7%, *p* = 0.80) and CPR (50.0% vs. 44.4%, *p* = 0.68) between PGT-A and conventional IVF/ICSI, while Mei et al. (37), similarly observed no significant difference in CPR (70% vs. 60%, *p* = 0.610). Consistent with these findings, Liu et al. (38), found no improvement in cumulative CPR after stratification by maternal age (< 38 years: 59.8% vs. 63.8%, *p* = 0.362; ≥38 years, 26.0% vs. 24.1%, *p* = 0.786).

A further layer of complexity is that improved early outcomes did not always translate into statistically significant improvements in later reproductive outcomes. Kim et al. (35), reported significantly higher IR (41.7% vs. 22.0%, *p* < 0.001), and CPR per ET cycle (61.0% vs. 44.4%, *p* = 0.004) in RIF patients undergoing PGT-A compared with controls, whereas ongoing pregnancy/LBR per clinical pregnancy did not differ significantly (77.0% vs. 64.4%, *p* = 0.085). Kato et al. (32), observed significant improvements in IR (75.0% vs. 33.2%, *p* < 0.0001) and CPR (73.2% vs. 27.2%, *p* < 0.0001)in the RIF protocol, whereas in a subsequent study (33), found no significant differences in IR or CPR within RIF subgroups (76.9% vs. 90%, *p* = 0.6036). Lee et al. (21), similarly reported that reproductive outcomes after PGT-A in RIF were not clearly superior to those observed in other high-risk indications, further emphasizing that RIF-specific benefit remain inconsistent across studies.

Taken together, these findings suggest that PGT-A may improve IR and CPR per transfer in selected RIF populations, particularly where embryo aneuploidy is likely to represent a major limiting factor. However, the absence of consistent benefit in uRIF/iRIF cohorts and the frequent dissociation between early and later endpoints indicate that IF cannot be explained by embryo ploidy alone. Rather, the available evidence supports a multifactorial model in which embryo competence, ER, uterine factors, and patient-specific characteristics jointly determine reproductive success.

### 3.4 Live birth rate

The pattern observed for LBR largely mirrors that seen for implantation and clinical pregnancy outcomes. Several studies reported significantly higher LBR per ET following PGT-A, whereas others observed numerically favorable but non-significant differences or comparable outcomes between groups. The evidence becomes more heterogeneous when LBR is assessed cumulatively or per patient.

Per-transfer analyses generally favored PGT-A. Sato et al. (22), reported a significantly higher LBR per ET in the PGT-A group compared with controls (62.5% vs. 31.7%, *p* = 0.016), and Pantou et al. (26), similarly observed higher live birth per ET among RIF patients undergoing PGT-A (47.8% vs. 19.0%, *p* = 0.015). In larger retrospective cohorts, Gu et al. (31), showed that the LBR advantage became statistically significant after propensity score matching (43.1% vs. 25.7%, *p* = 0.007), although the difference was not significant before propensity score matching (42.5% vs. 36.0%, *p* = 0.076). Likewise, Wang et al. (28), reported higher LBR per transfer in RIF + PGT-A compared with RIF + no PGT-A (47.6% vs. 31.4%, *p* = 0.018). Kim et al. (35), also observed significantly higher ongoing pregnancy/LBR per ET after PGT-A (47.0% vs. 28.6%, *p* < 0.001), whereas this difference was not significant when analyzed per total cycle (36.2% vs. 28.6%, *p* = 0.126).

The distinction between PGT-A alone and integrated embryo-endometrium strategies is particularly relevant for LBR. Kozyra et al. (34), reported a 1.5-fold higher odds of live birth in patients undergoing PGT-A alone; however, this difference was not statistical significance (*p* = 0.439). In contrast, combining PGT-A with individualized implantation window (IW) assessment was associated with a significantly higher likelihood of live birth (*p* = 0.026). These findings suggest that, in selected RIF populations, optimizing embryo selection alone may be insufficient unless endometrial timing is also addressed.

Despite these favorable findings, not all studies confirmed an LBR benefit. Kato et al. (33), found no significant difference in LBR per ET in RIF patients undergoing minimal stimulation IVF (69.2% vs. 90.0%, *p* = 0.3394), and Lee et al. (21), did not demonstrate a clear live birth advantage for RIF patients undergoing PGT-A compared with other high-risk IVF populations (40.2%, *p* = 0.332 per initiated cycle/51.6%, *p* = 0.941 per ET cycle).

The evidence becomes even more conflicting when LBR is assessed cumulatively. Liu et al. (38), in a large uRIF cohort, reported no significant improvement in CLBR after PGT-A, either in women younger than 38 years (49.7% vs. 57.7%, *p* = 0.081) or in those aged 38 years or older (14.0% vs. 19.5%, *p* = 0.384). Similarly, Sato et al. (22), showed that the higher LBR per ET did not translate into a significantly higher LBR per patient (35.7% vs. 26.0%, *p* = 0.26). Kato et al. (32), reported significant differences in cumulative outcomes between groups (20.3% vs. 68.9%, *p* < 0.0001), although these findings appeared to be driven primarily by specific patient subgroups, particularly women with AMA. In iRIF, Shi et al. (29), found no significant LBR with PGT-A compared with conventional IVF/ICSI (35.0% vs. 26.7%, *p* = 0.50).

Taken together, these findings emphasize an important clinical distinction: PGT-A may improve the probability of live birth per transfer in selected RIF populations, but it does not consistently increase the cumulative probability of live birth per patient. Therefore, the principal benefit of PGT-A may lie in improving treatment efficiency by reducing unsuccessful transfers or shortening the time to a viable pregnancy, rather than universally increasing absolute reproductive success across all RIF populations.

### 3.5 Miscarriage rate

Unlike implantation, clinical pregnancy, and per-transfer live birth outcomes, MR did not show a consistent improvement with PGT-A in RIF populations. Across most studies, reductions in miscarriage were absent, statistically non-significant, or restricted to specific contexts, despite the transfer of euploid embryo.

Several studies reported no significant difference in MR between PGT-A and non-PGT-A groups. Sato et al. (22), despite demonstrating higher CPR and LBR per ET with PGT-A, found comparable MR per clinical pregnancy (11.8% vs. 0%, *p* = 0.999). Gu et al. (31), similarly reported no reduction in MR either before propensity score matching (7.1% vs. 5.6%, *p* = 0.390) or after matching (6.4% vs. 5.5%, *p* = 0.775). Du et al. (30), also found no significant difference in spontaneous abortion rates between PGT-A and control groups (21.43% vs. 19.40%, *p* = 0.525), despite the significantly higher CPR observed in the PGT-A group.

This lack of a consistent miscarriage benefit was also evident in uRIF and iRIF cohorts. Shi et al. (29), reported similar MR between PGT-A and conventional IVF/ICSI (30.0% vs. 40.0%, *p* = 0.70), while Mei et al. (37), found comparable miscarriage-related outcomes in uRIF patients (28.6% vs. 33.3%, *p* = 0.838). Liu et al. (38), likewise observed no significant reduction in clinical pregnancy loss after PGT-A (16.8% vs. 11.1%, *p* = 0.133), including both first-trimester (10.9% vs. 8.1%, *p* = 0.386) and second-trimester loss (5.9% vs. 3.0%, *p* = 0.304). Age-stratified yielded similar results, with no significant differences in overall pregnancy loss or trimester-specific losses between PGT-A and conventional IVF/ICSI groups.

Other studies reinforced the same conclusion. Kim et al. (35), reported a numerically lower early pregnancy loss rate in RIF patients undergoing PGT-A (13.1% vs. 24.6%); however, this difference did not reach statistical significance (*p* = 0.073). Wang et al. (28), also found no significant reduction in MR in RIF + PGT-A compared with RIF + no PGT-A (9.1% vs. 19.6%, *p* = 0.276), while Pantou et al. (26), reported comparable early abortion rates (18.75% vs. 21.4%, *p* = 0.857). Tong et al. (24), similarly found no significant difference in MR per transfer (7.8% vs. 4.3%, *p* > 0.05), and Lee et al. (21), did not identify significant differences in miscarriage rates between RIF and other high-risk IVF indications. Fodina et al. (25), further reported that neither ERA alone nor ERA combined with PGT-A significantly influenced miscarriage-related outcomes, supporting the notion that improved pregnancy rates do not necessarily translate into reduced pregnancy loss.

Only limited evidence suggested a potential reduction in pregnancy loss under specific conditions. Gajjar et al. (36), reported a lower abortion rate when PGT-A was combined with OpERA (29.6% vs. 46.3%, *p* = 0.03339), suggesting that miscarriage-related outcomes may improve when embryo selection is integrated with endometrial optimization. Kato et al. (32, 33), also reported significant differences in MR between groups in some analyses (20.3% vs. 68.9%, *p* < 0.0001), although these findings were not reproduced consistently across all RIF subgroups and should be interpreted cautiously in light of study design, subgroup composition, and outcome definitions.

Collectively, these findings indicate that PGT-A does not consistently reduce MR in RIF populations. This apparent dissociation between improved implantation or clinical pregnancy outcomes and unchanged MR suggests that pregnancy loss after implantation may be influenced by mechanisms extending beyond embryo aneuploidy alone. ER, uterine environment, immunological factors, and systemic maternal contributors may therefore remain clinically relevant even after the transfer of euploid embryos.

### 3.6 Role of embryo ploidy in implantation success

Embryo ploidy consistently emerges as a major biological determinant of implantation competence in patients with RIF, providing a strong biological rationale for the use of PGT-A. Across multiple cohorts, the selection and transfer of euploid embryos have been associated with higher IR and CPR, and, in several studies, LBR per ET (22, 26, 28, 31, 35). These findings support the concept that chromosomal competence is an important prerequisite for successful implantation and early pregnancy establishment.

However, the available evidence also indicates that euploidy alone does not fully explain implantation success. Persistent IF following transfer of euploid embryos has been documented in several RIF populations, including both younger and advanced-age cohorts (24, 29, 37, 38). Moreover, even in studies where PGT-A improved early reproductive endpoints, such as IR or CPR, downstream outcomes including ongoing pregnancy, miscarriage, or cumulative live birth often remain unchanged (30).These observations suggest that eliminating aneuploid embryos does not fully address the biological complexity underlying IF.

This apparent dissociation between improved embryo selection and inconsistent overall reproductive success is further supported by studies demonstrating comparable outcomes between PGT-A and conventional IVF/ICSI strategies in specific RIF subgroups (21, 33). Similarly, although PGT-A has been associated with improved live birth per transfer, cumulative outcomes at the patient level remain variable and frequently unchanged, particularly in unexplained RIF populations or in patients without a clearly increased aneuploidy burden (32, 33). Therefore, embryo ploidy appears to represent an important, but not exclusive, determinant of reproductive success.

Importantly, the relationship between embryo ploidy and implantation is likely modulated by additional biological and clinical factors. Several studies indicate that embryo competence interacts with ER and uterine environment, rather than acting as an isolated determinant of success (23, 25, 27, 34, 36). This concept is supported by studies demonstrating that combining embryo selection with endometrial optimization strategies, such as ERA-guided or IW-based approaches, may yield superior outcomes compared with PGT-A alone in selected RIF populations.

Collectively, these findings support a multifactorial model of IF in RIF. While embryo aneuploidy constitutes an important limiting factor and a biologically plausible therapeutic target, it does not appear to be the sole or dominant mechanism in all patients. Instead, successful implantation likely depends on the interplay among embryo chromosomal integrity, ER, uterine factors, immunological mechanisms, and broader patient-specific characteristics. Consequently, the clinical utility of PGT-A in RIF should be interpreted within this broader biological context, rather than as a universally effective standalone intervention.

Taken together, the current evidence suggests that embryo ploidy is a necessary, but not sufficient, condition for successful implantation in RIF. Future studies should therefore move beyond a binary view of embryo euploidy and explore integrated models that simultaneously account for embryo, endometrial, and maternal factors in order to better define the patients most likely to benefit from PGT-A.

## 4 Discussion

The present scoping review synthesizes evidence from 18 studies evaluating the role of PGT-A in RIF and highlights a consistent yet nuanced pattern: while PGT-A may improve reproductive outcomes at the level of individual ET, its impact on cumulative reproductive success and miscarriage reduction remains inconsistent.

The methodological quality of the included studies was generally moderate to high according to the NOS; however, methodological quality should not be interpreted as evidence certainty. Most studies were retrospective observational cohorts characterized by substantial heterogeneity in RIF definitions, patient selection, PGT-A methodologies, and reported outcomes. Consequently, the favorable findings observed in some cohorts should be interpreted as hypothesis-generating rather than definitive evidence supporting the routine use of PGT-A in all RIF populations.

Across the included studies, a relatively coherent signal emerged regarding per-transfer outcomes. Multiple cohorts demonstrated that transfer of euploid embryos is associated with higher IR, CPR, and, in some studies, LBR per ET, supporting the biological premise that embryo chromosomal competence is an important determinant of early reproductive success (22, 26, 28, 30, 31, 35). These observations are consistent with recent meta-analyses, which reported significantly improved IR, CPR, and LBR following PGT-A, particularly when outcomes were assessed per transfer rather than per patient (9, 10).

However, the apparent benefit of PGT-A becomes less consistent when outcomes are evaluated cumulatively. Several studies, particularly those focusing on uRIF or iRIF populations, failed to demonstrate meaningful improvements in CLBR or other patient-level outcomes (29, 37, 38). Similar findings were reported by Tong et al. (24), suggesting that once embryo ploidy is controlled, additional biological and clinical factors may become limiting.

These observations are also supported by broader systematic reviews showing that improvements in transfer efficiency do not necessarily translate into higher LBR per patient (11).

This discrepancy between per-transfer efficiency and cumulative effectiveness represents one of the central findings of both this review and the broader literature. PGT-A appears to function primarily as an embryo selection tool that increases the probability of success in a given transfer rather than universally increasing the overall likelihood of achieving a live birth throughout an entire treatment course.

This interpretation is further supported by miscarriage outcomes. Across most included studies, miscarriage rates were comparable between PGT-A and non-PGT-A groups despite improved implantation and clinical pregnancy outcomes. Although some studies and meta-analyses reported modest reductions in pregnancy loss, these findings were not consistently reproduced in RIF-specific cohorts (9, 21, 25, 27, 29, 30, 34, 37). Therefore, embryo euploidy alone does not appear to fully explain the persistence of miscarriage and IF in RIF.

A plausible explanation for these inconsistencies lies in the heterogeneous pathophysiology of RIF. The available evidence suggests that the effectiveness of PGT-A varies according to the relative contribution of embryo-related and non-embryonic factors. In populations characterized by a higher risk of embryo aneuploidy, such as AMA or repeated failure after transfer of untested embryos, PGT-A appears to confer greater clinical benefit (22, 28, 31). Conversely, in uRIF and iRIF, reproductive outcomes are often comparable between PGT-A and conventional IVF/ICSI (29, 37, 38), suggesting that embryo ploidy alone cannot account for IF.

The interaction between embryo competence and ER further complicates this relationship. Several studies included in this review suggest that combined strategies integrating PGT-A with personalized endometrial approaches may improve reproductive outcomes in selected patients (23, 27, 34, 36), although the available evidence remains inconsistent. These findings support the concept that implantation success depends on embryo-endometrium synchrony, rather than embryo competence alone.

Compared with previous systematic reviews and meta-analyses, this scoping review provides several additional contributions. First, it focuses specifically on RIF as a distinct clinical entity rather than extrapolating from heterogeneous IVF populations. Second, it emphasizes the distinction between per-transfer and cumulative outcomes, highlighting that treatment efficiency and overall reproductive success are not necessarily equivalent. Third, it proposes a conceptual framework that stratifies RIF into embryo- and endometrium-driven, and mixed phenotypes, thereby providing a biologically plausible explanation for the heterogeneous clinical effectiveness of PGT-A across patient populations.

**Figure 3:**
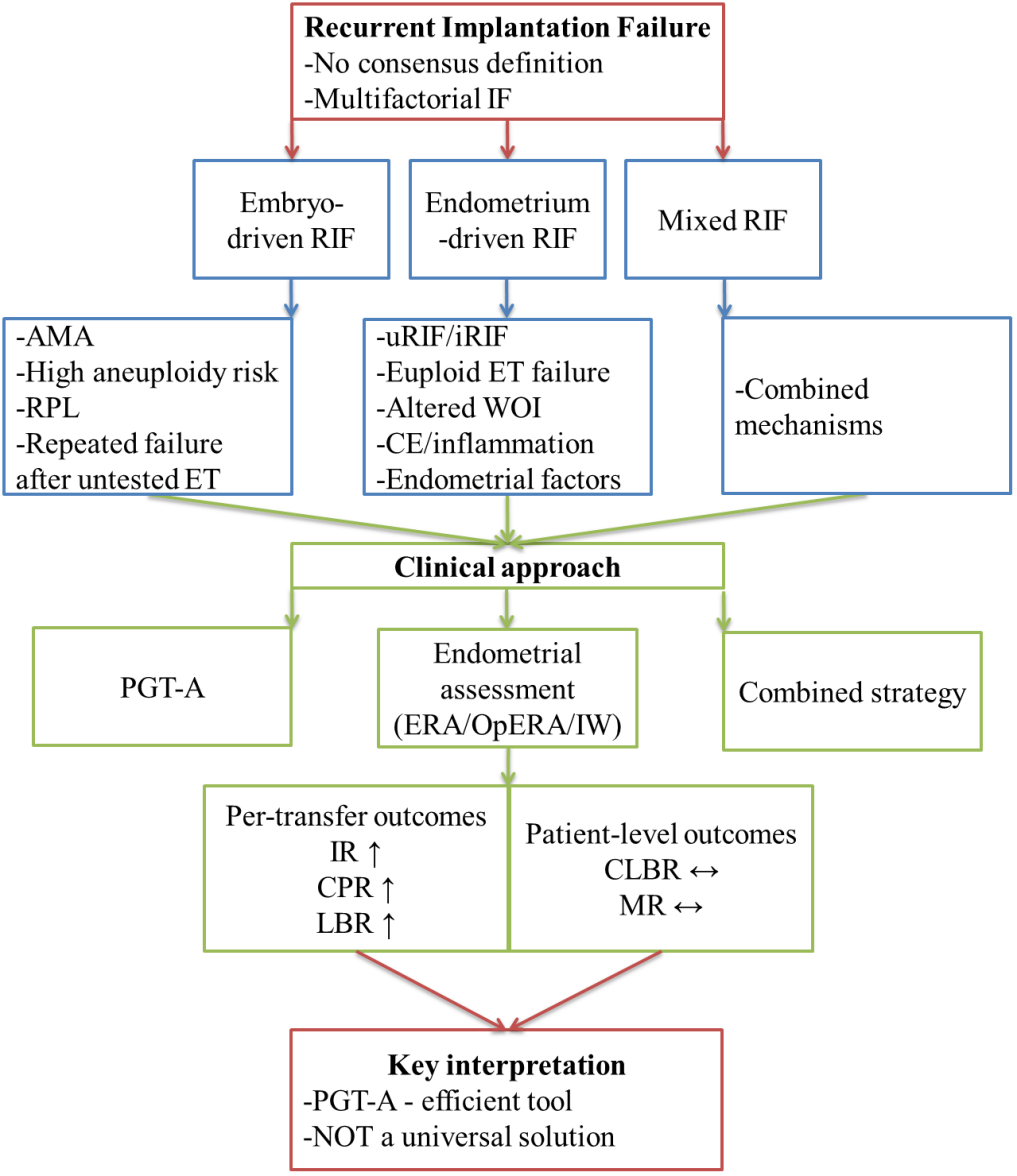
Proposed phenotype-based framework for PGT-A use in RIF. The figure illustrates a conceptual model stratifying RIF into embryo-driven, endometrium-driven, and mixed phenotypes. The framework highlights how underlying biological mechanisms may influence the effectiveness of PGT-A and explains why improvements in per-transfer outcomes (IR, CPR, and LBR) do not necessarily translate into consistent improvements in patient-level outcomes, such as CLBR or MR. PGT-A is therefore proposed as an efficient embryo-selection strategy that may benefit selected RIF phenotypes rather than a universal intervention.

Taken together, the findings of this review suggest that PGT-A improves treatment efficiency rather than absolute reproductive success. While it may increase the probability of implantation and live birth per transfer in selected patients, it does not consistently improve CLBR or reduce miscarriage risk. As illustrated in **Figure 3**, embryo ploidy appears to be a necessary, but not sufficient, condition for successful implantation in RIF. Consequently, the clinical utility of PGT-A should be interpreted within a broader multifactorial framework that incorporates embryo competence, ER, uterine environment, and patient-specific characteristics, ultimately supporting a more individualized approach to the management of RIF.

Considerable methodological heterogeneity exists across the included studies with respect to embryo quality assessment, mosaicism classification, transfer policies, and freeze-all strategies (**Table 3**). To integrate these findings and provide a clinically oriented interpretation, we propose a conceptual framework distinguishing embryo-driven, endometrium-driven, and mixed forms of RIF, highlighting how these phenotypes may influence the clinical utility of PGT-A (**Figure 3**).

**Table 3:** Embryo quality criteria and PGT-A laboratory characteristics in studies evaluating RIF.

Study	Embryo quality	Mosaicism	Transfer type	Freeze-all
(Lee et al., 2019) (21)	Morphological (blastocyst stage)	NS	FET (euploid blastocysts)	Yes
(Sato et al., 2019) (22)	Morphological selection (best-quality blastocyst)	Partially reported (suspected mosaicism)	Single FET (euploid prioritized)	Yes
(Cozzolino et al., 2020) (23)	Good-quality (ICM/TE grading, D5 blastocysts)	NS	Fresh + FET	Partial
(Tong et al., 2021) (24)	Good-quality	Defined (NGS thresholds: 20-50%)	Single FET (euploid embryo)	Yes
(Fodina et al., 2021) (25)	Good-quality	Defined (NGS thresholds: 20-50%, 50-80%)	FET	Yes
(Pantou et al., 2022) (26)	High-quality (expanded blastocysts, ICM/TE grading)	NS	Fresh ET (1–3 embryos)	No
(Amin et al., 2022) (27)	Morphological/euploid embryos	Defined (NGS thresholds: <20/20-80/>80)	PET (ERA-guided FET)	NS
(Wang et al., 2023) (28)	Good-quality (≥4BC, Gardner criteria)	Partially reported (suspected mosaicism excluded)	Single FET (euploid blastocyst)	Yes
(Shi et al., 2023) (29)	Good-quality	Defined (NGS thresholds: 30-70%)	Single ET (euploid embryo)	NS
(Du et al., 2023) (30)	Good-quality (AA-BB defined)	Defined (NGS thresholds: <20/21–80/>80)	Single FET (blastocyst)	Yes
(Gu et al., 2023) (31)	Good-quality (≥B, Gardner criteria)	NS	FET (SET ± DET blastocysts)	Yes
(Kato et al., 2023a) (32)	High-quality (ICM/TE ≥B, Gardner criteria)	Partially reported (euploid with suspected mosaicism)	SVBT	Yes
(Kato et al., 2023b) (33)	Good-quality (ICM/TE ≥ B, Gardner criteria)	Reported (euploid + suspected mosaic)	SVBT	Yes
(Kozyra et al., 2024) (34)	High-quality (AA, AB, VA, BB)	NS	FET	Yes
(Kim et al., 2024) (35)	Morphological (blastocyst stage)	Reported (no-call embryos)	FET (1–2 euploid embryos)	Yes
(Gajjar et al., 2024) (36)	Good-quality (≥4BB, Gardner criteria)	Defined (NGS thresholds: <20/20-80/>80)	FET	NS
(Mei et al., 2024) (37)	NS	NS	Single FET (euploid blastocyst)	Yes
(Liu et al., 2024) (38)	Good-quality (≥4BC, Gardner criteria)	NS	Single FET (euploid embryo)	Yes

NS, not specified; FET, frozen embryo transfer; ICM, inner cell mass; TE, trophectoderm; NGS, next-generation sequencing; ET, embryo transfer; PET, personalized ET; ERA, endometrial receptivity assay; SET, single ET; DET, double ET; SVBT, single vitrified-warmed blastocyst transfer.

### 4.1 Current controversies and unresolved issues in PGT-A

Despite the growing adoption of PGT-A, several important controversies remain unresolved and should be considered when interpreting the available evidence. One of the most debated issues concerns the clinical management of mosaic embryos. Advances in NGS have increased the detection of embryonic mosaicism; however, the predictive value of mosaic findings remains uncertain because mosaic embryos may still result in healthy live births. Consequently, the exclusion of mosaic embryos may reduce the number of transferable embryos and potentially affect cumulative reproductive outcomes.

Another area of ongoing debate relates to the invasive nature of TE biopsy. Although contemporary studies generally suggest that blastocyst-stage biopsy has minimal impact on embryo viability when performed in experienced centers, concerns remain regarding potential biopsy-related harm, particularly in embryos with limited developmental potential or when laboratory expertise varies substantially.

In addition, false-positive aneuploidy findings represent a recognized limitation of current PGT-A technologies. Technical factors, sampling bias, and biological discordance between TE and ICM may lead to misclassification of embryos, raising concerns that embryos with reproductive potential could be unnecessarily discarded. These limitations are particularly relevant when interpreting studies reporting improvements in per-transfer outcomes without corresponding gains in cumulative live birth rates.

Taken together, these controversies emphasize that PGT-A should not be regarded as a universally applicable intervention. Instead, its clinical value is likely to depend on careful patient selection, laboratory expertise, and a comprehensive understanding of both embryonic and non-embryonic contributors to implantation success.

## 5 Conclusions

This scoping review suggests that PGT-A may improve reproductive efficiency in selected patients with RIF, primarily by increasing IR, CPR, and LBR per ET. However, these benefits do not consistently translate into improved CLBR or reduced MR.

The available evidence indicates that embryo ploidy is an important determinant of implantation potential, but it is unlikely to fully explain RIF. Persistent IF following euploid ET supports a multifactorial model in which embryo competence interacts with ER, uterine environment, immunological factors, and patient-specific factors.

Accordingly, we proposed a conceptual framework distinguishing embryo-driven, endometrium-driven, and mixed forms of RIF. Within this framework, the clinical utility of PGT-A appears to be context-dependent, with greater potential benefit in patients with a higher likelihood of embryo-related failure, such as those of AMA or with an increased risk of aneuploidy, whereas its routine application in unselected, uRIF, or iRIF populations remains difficult to justify. Future research should prioritize prospective studies using standardized RIF definitions, harmonized laboratory protocols, and comprehensive reporting of both per-transfer and cumulative outcomes. Integrated approaches simultaneously assessing embryo competence and endometrial factors may further refine patient selection and help define the optimal clinical role of PGT-A in RIF.

## Data Availability

The original contributions presented in the study are included in the article/**Supplementary Material**. Further inquiries can be directed to the corresponding author.
