# Supplementary material for: Mapping the evidence and research gaps on preimplantation genetic testing for aneuploidy in recurrent implantation failure: a scoping review
Source: Front Endocrinol (Lausanne). 2026 Jul 27;17:1872860. doi: 10.3389/fendo.2026.1872860 (PMC13453691; doi:10.3389/fendo.2026.1872860)
Supplement: Supplementary file 1 [file SupplementaryFile1.docx]

**Supplementary File 1.** Detailed searching strategies

**Primary keywords**

Recurrent Implantation Failure, RIF, Repeated Implantation Failure, Preimplantation Genetic Testing, Pre-implantation Genetic Testing, Aneuploidy.

**1. PubMed-MEDLINE – United States (NLM, 1996)**

*1.1. Extensive searching string:*

| **#1** | **(((((Recurrent Implantation Failure) OR (RIF)) OR (Repeated Implantation Failure)) AND (Preimplantation Genetic Testing)) OR (Pre-implantation Genetic Testing)) AND (Aneuploidy)**  ((((("recurrance"[All Fields] OR "recurrence"[MeSH Terms] OR "recurrence"[All Fields] OR "recurrences"[All Fields] OR "recurrencies"[All Fields] OR "recurrency"[All Fields] OR "recurrent"[All Fields] OR "recurrently"[All Fields] OR "recurrents"[All Fields]) AND ("drug implants"[Supplementary Concept] OR "drug implants"[All Fields] OR "implant"[All Fields] OR "drug implants"[MeSH Terms] OR ("drug"[All Fields] AND "implants"[All Fields]) OR "embryo implantation"[MeSH Terms] OR ("embryo"[All Fields] AND "implantation"[All Fields]) OR "embryo implantation"[All Fields] OR "implantation"[All Fields] OR "implant s"[All Fields] OR "implantability"[All Fields] OR "implantable"[All Fields] OR "implantables"[All Fields] OR "implantate"[All Fields] OR "implantated"[All Fields] OR "implantates"[All Fields] OR "implantations"[All Fields] OR "implanted"[All Fields] OR "implanter"[All Fields] OR "implanters"[All Fields] OR "implanting"[All Fields] OR "implantion"[All Fields] OR "implantitis"[All Fields] OR "implants"[All Fields]) AND ("failure"[All Fields] OR "failures"[All Fields])) OR ("rev infect"[Journal] OR "rif"[All Fields]) OR (("repeatabilities"[All Fields] OR "repeatability"[All Fields] OR "repeatable"[All Fields] OR "repeated"[All Fields] OR "repeatibility"[All Fields]) AND ("drug implants"[Supplementary Concept] OR "drug implants"[All Fields] OR "implant"[All Fields] OR "drug implants"[MeSH Terms] OR ("drug"[All Fields] AND "implants"[All Fields]) OR "embryo implantation"[MeSH Terms] OR ("embryo"[All Fields] AND "implantation"[All Fields]) OR "embryo implantation"[All Fields] OR "implantation"[All Fields] OR "implant s"[All Fields] OR "implantability"[All Fields] OR "implantable"[All Fields] OR "implantables"[All Fields] OR "implantate"[All Fields] OR "implantated"[All Fields] OR "implantates"[All Fields] OR "implantations"[All Fields] OR "implanted"[All Fields] OR "implanter"[All Fields] OR "implanters"[All Fields] OR "implanting"[All Fields] OR "implantion"[All Fields] OR "implantitis"[All Fields] OR "implants"[All Fields]) AND ("failure"[All Fields] OR "failures"[All Fields]))) AND (("preimplantation"[All Fields] OR "preimplantational"[All Fields] OR "preimplantion"[All Fields]) AND ("genetic testing"[MeSH Terms] OR ("genetic"[All Fields] AND "testing"[All Fields]) OR "genetic testing"[All Fields]))) OR ("Pre-implantation"[All Fields] AND ("genetic testing"[MeSH Terms] OR ("genetic"[All Fields] AND "testing"[All Fields]) OR "genetic testing"[All Fields]))) AND ("aneuploidy"[MeSH Terms] OR "aneuploidy"[All Fields] OR "aneuploidies"[All Fields]) | *231* |
| --- | --- | --- |
| **#2** | **(((((Recurrent Implantation Failure) OR (RIF)) OR (Repeated Implantation Failure)) AND (Preimplantation Genetic Testing)) OR (Pre-implantation Genetic Testing)) AND (Aneuploidy) Filters: from 2016/4/1 - 2026/4/1**  (((((("recurrance"[All Fields] OR "recurrence"[MeSH Terms] OR "recurrence"[All Fields] OR "recurrences"[All Fields] OR "recurrencies"[All Fields] OR "recurrency"[All Fields] OR "recurrent"[All Fields] OR "recurrently"[All Fields] OR "recurrents"[All Fields]) AND ("drug implants"[Supplementary Concept] OR "drug implants"[All Fields] OR "implant"[All Fields] OR "drug implants"[MeSH Terms] OR ("drug"[All Fields] AND "implants"[All Fields]) OR "embryo implantation"[MeSH Terms] OR ("embryo"[All Fields] AND "implantation"[All Fields]) OR "embryo implantation"[All Fields] OR "implantation"[All Fields] OR "implant s"[All Fields] OR "implantability"[All Fields] OR "implantable"[All Fields] OR "implantables"[All Fields] OR "implantate"[All Fields] OR "implantated"[All Fields] OR "implantates"[All Fields] OR "implantations"[All Fields] OR "implanted"[All Fields] OR "implanter"[All Fields] OR "implanters"[All Fields] OR "implanting"[All Fields] OR "implantion"[All Fields] OR "implantitis"[All Fields] OR "implants"[All Fields]) AND ("failure"[All Fields] OR "failures"[All Fields])) OR ("rev infect"[Journal] OR "rif"[All Fields]) OR (("repeatabilities"[All Fields] OR "repeatability"[All Fields] OR "repeatable"[All Fields] OR "repeated"[All Fields] OR "repeatibility"[All Fields]) AND ("drug implants"[Supplementary Concept] OR "drug implants"[All Fields] OR "implant"[All Fields] OR "drug implants"[MeSH Terms] OR ("drug"[All Fields] AND "implants"[All Fields]) OR "embryo implantation"[MeSH Terms] OR ("embryo"[All Fields] AND "implantation"[All Fields]) OR "embryo implantation"[All Fields] OR "implantation"[All Fields] OR "implant s"[All Fields] OR "implantability"[All Fields] OR "implantable"[All Fields] OR "implantables"[All Fields] OR "implantate"[All Fields] OR "implantated"[All Fields] OR "implantates"[All Fields] OR "implantations"[All Fields] OR "implanted"[All Fields] OR "implanter"[All Fields] OR "implanters"[All Fields] OR "implanting"[All Fields] OR "implantion"[All Fields] OR "implantitis"[All Fields] OR "implants"[All Fields]) AND ("failure"[All Fields] OR "failures"[All Fields]))) AND (("preimplantation"[All Fields] OR "preimplantational"[All Fields] OR "preimplantion"[All Fields]) AND ("genetic testing"[MeSH Terms] OR ("genetic"[All Fields] AND "testing"[All Fields]) OR "genetic testing"[All Fields]))) OR ("Pre-implantation"[All Fields] AND ("genetic testing"[MeSH Terms] OR ("genetic"[All Fields] AND "testing"[All Fields]) OR "genetic testing"[All Fields]))) AND ("aneuploidy"[MeSH Terms] OR "aneuploidy"[All Fields] OR "aneuploidies"[All Fields])) AND (2016/4/1:2026/4/1[pdat]) | *196* |
| **#3** | **(((((Recurrent Implantation Failure) OR (RIF)) OR (Repeated Implantation Failure)) AND (Preimplantation Genetic Testing)) OR (Pre-implantation Genetic Testing)) AND (Aneuploidy) Filters: English, from 2016/4/1 - 2026/4/1**  (((((("recurrance"[All Fields] OR "recurrence"[MeSH Terms] OR "recurrence"[All Fields] OR "recurrences"[All Fields] OR "recurrencies"[All Fields] OR "recurrency"[All Fields] OR "recurrent"[All Fields] OR "recurrently"[All Fields] OR "recurrents"[All Fields]) AND ("drug implants"[Supplementary Concept] OR "drug implants"[All Fields] OR "implant"[All Fields] OR "drug implants"[MeSH Terms] OR ("drug"[All Fields] AND "implants"[All Fields]) OR "embryo implantation"[MeSH Terms] OR ("embryo"[All Fields] AND "implantation"[All Fields]) OR "embryo implantation"[All Fields] OR "implantation"[All Fields] OR "implant s"[All Fields] OR "implantability"[All Fields] OR "implantable"[All Fields] OR "implantables"[All Fields] OR "implantate"[All Fields] OR "implantated"[All Fields] OR "implantates"[All Fields] OR "implantations"[All Fields] OR "implanted"[All Fields] OR "implanter"[All Fields] OR "implanters"[All Fields] OR "implanting"[All Fields] OR "implantion"[All Fields] OR "implantitis"[All Fields] OR "implants"[All Fields]) AND ("failure"[All Fields] OR "failures"[All Fields])) OR ("rev infect"[Journal] OR "rif"[All Fields]) OR (("repeatabilities"[All Fields] OR "repeatability"[All Fields] OR "repeatable"[All Fields] OR "repeated"[All Fields] OR "repeatibility"[All Fields]) AND ("drug implants"[Supplementary Concept] OR "drug implants"[All Fields] OR "implant"[All Fields] OR "drug implants"[MeSH Terms] OR ("drug"[All Fields] AND "implants"[All Fields]) OR "embryo implantation"[MeSH Terms] OR ("embryo"[All Fields] AND "implantation"[All Fields]) OR "embryo implantation"[All Fields] OR "implantation"[All Fields] OR "implant s"[All Fields] OR "implantability"[All Fields] OR "implantable"[All Fields] OR "implantables"[All Fields] OR "implantate"[All Fields] OR "implantated"[All Fields] OR "implantates"[All Fields] OR "implantations"[All Fields] OR "implanted"[All Fields] OR "implanter"[All Fields] OR "implanters"[All Fields] OR "implanting"[All Fields] OR "implantion"[All Fields] OR "implantitis"[All Fields] OR "implants"[All Fields]) AND ("failure"[All Fields] OR "failures"[All Fields]))) AND (("preimplantation"[All Fields] OR "preimplantational"[All Fields] OR "preimplantion"[All Fields]) AND ("genetic testing"[MeSH Terms] OR ("genetic"[All Fields] AND "testing"[All Fields]) OR "genetic testing"[All Fields]))) OR ("Pre-implantation"[All Fields] AND ("genetic testing"[MeSH Terms] OR ("genetic"[All Fields] AND "testing"[All Fields]) OR "genetic testing"[All Fields]))) AND ("aneuploidy"[MeSH Terms] OR "aneuploidy"[All Fields] OR "aneuploidies"[All Fields])) AND ((2016/4/1:2026/4/1[pdat]) AND (english[Filter])) | *195* |
| **#4** | **(((((Recurrent Implantation Failure) OR (RIF)) OR (Repeated Implantation Failure)) AND (Preimplantation Genetic Testing)) OR (Pre-implantation Genetic Testing)) AND (Aneuploidy) Filters: English, Exclude preprints, from 2016/4/1 - 2026/4/1**  (((((("recurrance"[All Fields] OR "recurrence"[MeSH Terms] OR "recurrence"[All Fields] OR "recurrences"[All Fields] OR "recurrencies"[All Fields] OR "recurrency"[All Fields] OR "recurrent"[All Fields] OR "recurrently"[All Fields] OR "recurrents"[All Fields]) AND ("drug implants"[Supplementary Concept] OR "drug implants"[All Fields] OR "implant"[All Fields] OR "drug implants"[MeSH Terms] OR ("drug"[All Fields] AND "implants"[All Fields]) OR "embryo implantation"[MeSH Terms] OR ("embryo"[All Fields] AND "implantation"[All Fields]) OR "embryo implantation"[All Fields] OR "implantation"[All Fields] OR "implant s"[All Fields] OR "implantability"[All Fields] OR "implantable"[All Fields] OR "implantables"[All Fields] OR "implantate"[All Fields] OR "implantated"[All Fields] OR "implantates"[All Fields] OR "implantations"[All Fields] OR "implanted"[All Fields] OR "implanter"[All Fields] OR "implanters"[All Fields] OR "implanting"[All Fields] OR "implantion"[All Fields] OR "implantitis"[All Fields] OR "implants"[All Fields]) AND ("failure"[All Fields] OR "failures"[All Fields])) OR ("rev infect"[Journal] OR "rif"[All Fields]) OR (("repeatabilities"[All Fields] OR "repeatability"[All Fields] OR "repeatable"[All Fields] OR "repeated"[All Fields] OR "repeatibility"[All Fields]) AND ("drug implants"[Supplementary Concept] OR "drug implants"[All Fields] OR "implant"[All Fields] OR "drug implants"[MeSH Terms] OR ("drug"[All Fields] AND "implants"[All Fields]) OR "embryo implantation"[MeSH Terms] OR ("embryo"[All Fields] AND "implantation"[All Fields]) OR "embryo implantation"[All Fields] OR "implantation"[All Fields] OR "implant s"[All Fields] OR "implantability"[All Fields] OR "implantable"[All Fields] OR "implantables"[All Fields] OR "implantate"[All Fields] OR "implantated"[All Fields] OR "implantates"[All Fields] OR "implantations"[All Fields] OR "implanted"[All Fields] OR "implanter"[All Fields] OR "implanters"[All Fields] OR "implanting"[All Fields] OR "implantion"[All Fields] OR "implantitis"[All Fields] OR "implants"[All Fields]) AND ("failure"[All Fields] OR "failures"[All Fields]))) AND (("preimplantation"[All Fields] OR "preimplantational"[All Fields] OR "preimplantion"[All Fields]) AND ("genetic testing"[MeSH Terms] OR ("genetic"[All Fields] AND "testing"[All Fields]) OR "genetic testing"[All Fields]))) OR ("Pre-implantation"[All Fields] AND ("genetic testing"[MeSH Terms] OR ("genetic"[All Fields] AND "testing"[All Fields]) OR "genetic testing"[All Fields]))) AND ("aneuploidy"[MeSH Terms] OR "aneuploidy"[All Fields] OR "aneuploidies"[All Fields])) AND ((excludepreprints[Filter]) AND (2016/4/1:2026/4/1[pdat]) AND (english[Filter])) | *195* |
| **#5** | **(((((Recurrent Implantation Failure) OR (RIF)) OR (Repeated Implantation Failure)) AND (Preimplantation Genetic Testing)) OR (Pre-implantation Genetic Testing)) AND (Aneuploidy) Filters: English, Exclude preprints, MEDLINE, from 2016/4/1 - 2026/4/1**  (((((("recurrance"[All Fields] OR "recurrence"[MeSH Terms] OR "recurrence"[All Fields] OR "recurrences"[All Fields] OR "recurrencies"[All Fields] OR "recurrency"[All Fields] OR "recurrent"[All Fields] OR "recurrently"[All Fields] OR "recurrents"[All Fields]) AND ("drug implants"[Supplementary Concept] OR "drug implants"[All Fields] OR "implant"[All Fields] OR "drug implants"[MeSH Terms] OR ("drug"[All Fields] AND "implants"[All Fields]) OR "embryo implantation"[MeSH Terms] OR ("embryo"[All Fields] AND "implantation"[All Fields]) OR "embryo implantation"[All Fields] OR "implantation"[All Fields] OR "implant s"[All Fields] OR "implantability"[All Fields] OR "implantable"[All Fields] OR "implantables"[All Fields] OR "implantate"[All Fields] OR "implantated"[All Fields] OR "implantates"[All Fields] OR "implantations"[All Fields] OR "implanted"[All Fields] OR "implanter"[All Fields] OR "implanters"[All Fields] OR "implanting"[All Fields] OR "implantion"[All Fields] OR "implantitis"[All Fields] OR "implants"[All Fields]) AND ("failure"[All Fields] OR "failures"[All Fields])) OR ("rev infect"[Journal] OR "rif"[All Fields]) OR (("repeatabilities"[All Fields] OR "repeatability"[All Fields] OR "repeatable"[All Fields] OR "repeated"[All Fields] OR "repeatibility"[All Fields]) AND ("drug implants"[Supplementary Concept] OR "drug implants"[All Fields] OR "implant"[All Fields] OR "drug implants"[MeSH Terms] OR ("drug"[All Fields] AND "implants"[All Fields]) OR "embryo implantation"[MeSH Terms] OR ("embryo"[All Fields] AND "implantation"[All Fields]) OR "embryo implantation"[All Fields] OR "implantation"[All Fields] OR "implant s"[All Fields] OR "implantability"[All Fields] OR "implantable"[All Fields] OR "implantables"[All Fields] OR "implantate"[All Fields] OR "implantated"[All Fields] OR "implantates"[All Fields] OR "implantations"[All Fields] OR "implanted"[All Fields] OR "implanter"[All Fields] OR "implanters"[All Fields] OR "implanting"[All Fields] OR "implantion"[All Fields] OR "implantitis"[All Fields] OR "implants"[All Fields]) AND ("failure"[All Fields] OR "failures"[All Fields]))) AND (("preimplantation"[All Fields] OR "preimplantational"[All Fields] OR "preimplantion"[All Fields]) AND ("genetic testing"[MeSH Terms] OR ("genetic"[All Fields] AND "testing"[All Fields]) OR "genetic testing"[All Fields]))) OR ("Pre-implantation"[All Fields] AND ("genetic testing"[MeSH Terms] OR ("genetic"[All Fields] AND "testing"[All Fields]) OR "genetic testing"[All Fields]))) AND ("aneuploidy"[MeSH Terms] OR "aneuploidy"[All Fields] OR "aneuploidies"[All Fields])) AND ((excludepreprints[Filter] OR medline[Filter]) AND (2016/4/1:2026/4/1[pdat]) AND (english[Filter])) | *195* |
| **#6** | **(((((Recurrent Implantation Failure) OR (RIF)) OR (Repeated Implantation Failure)) AND (Preimplantation Genetic Testing)) OR (Pre-implantation Genetic Testing)) AND (Aneuploidy) Filters: English, Humans, Exclude preprints, MEDLINE, from 2016/4/1 - 2026/4/1**  (((((("recurrance"[All Fields] OR "recurrence"[MeSH Terms] OR "recurrence"[All Fields] OR "recurrences"[All Fields] OR "recurrencies"[All Fields] OR "recurrency"[All Fields] OR "recurrent"[All Fields] OR "recurrently"[All Fields] OR "recurrents"[All Fields]) AND ("drug implants"[Supplementary Concept] OR "drug implants"[All Fields] OR "implant"[All Fields] OR "drug implants"[MeSH Terms] OR ("drug"[All Fields] AND "implants"[All Fields]) OR "embryo implantation"[MeSH Terms] OR ("embryo"[All Fields] AND "implantation"[All Fields]) OR "embryo implantation"[All Fields] OR "implantation"[All Fields] OR "implant s"[All Fields] OR "implantability"[All Fields] OR "implantable"[All Fields] OR "implantables"[All Fields] OR "implantate"[All Fields] OR "implantated"[All Fields] OR "implantates"[All Fields] OR "implantations"[All Fields] OR "implanted"[All Fields] OR "implanter"[All Fields] OR "implanters"[All Fields] OR "implanting"[All Fields] OR "implantion"[All Fields] OR "implantitis"[All Fields] OR "implants"[All Fields]) AND ("failure"[All Fields] OR "failures"[All Fields])) OR ("rev infect"[Journal] OR "rif"[All Fields]) OR (("repeatabilities"[All Fields] OR "repeatability"[All Fields] OR "repeatable"[All Fields] OR "repeated"[All Fields] OR "repeatibility"[All Fields]) AND ("drug implants"[Supplementary Concept] OR "drug implants"[All Fields] OR "implant"[All Fields] OR "drug implants"[MeSH Terms] OR ("drug"[All Fields] AND "implants"[All Fields]) OR "embryo implantation"[MeSH Terms] OR ("embryo"[All Fields] AND "implantation"[All Fields]) OR "embryo implantation"[All Fields] OR "implantation"[All Fields] OR "implant s"[All Fields] OR "implantability"[All Fields] OR "implantable"[All Fields] OR "implantables"[All Fields] OR "implantate"[All Fields] OR "implantated"[All Fields] OR "implantates"[All Fields] OR "implantations"[All Fields] OR "implanted"[All Fields] OR "implanter"[All Fields] OR "implanters"[All Fields] OR "implanting"[All Fields] OR "implantion"[All Fields] OR "implantitis"[All Fields] OR "implants"[All Fields]) AND ("failure"[All Fields] OR "failures"[All Fields]))) AND (("preimplantation"[All Fields] OR "preimplantational"[All Fields] OR "preimplantion"[All Fields]) AND ("genetic testing"[MeSH Terms] OR ("genetic"[All Fields] AND "testing"[All Fields]) OR "genetic testing"[All Fields]))) OR ("Pre-implantation"[All Fields] AND ("genetic testing"[MeSH Terms] OR ("genetic"[All Fields] AND "testing"[All Fields]) OR "genetic testing"[All Fields]))) AND ("aneuploidy"[MeSH Terms] OR "aneuploidy"[All Fields] OR "aneuploidies"[All Fields])) AND ((excludepreprints[Filter] OR medline[Filter]) AND (humans[Filter]) AND (2016/4/1:2026/4/1[pdat]) AND (english[Filter])) | *155* |
| **#7** | **(((((Recurrent Implantation Failure) OR (RIF)) OR (Repeated Implantation Failure)) AND (Preimplantation Genetic Testing)) OR (Pre-implantation Genetic Testing)) AND (Aneuploidy) Filters: English, Humans, Female, Exclude preprints, MEDLINE, from 2016/4/1 - 2026/4/1**  (((((("recurrance"[All Fields] OR "recurrence"[MeSH Terms] OR "recurrence"[All Fields] OR "recurrences"[All Fields] OR "recurrencies"[All Fields] OR "recurrency"[All Fields] OR "recurrent"[All Fields] OR "recurrently"[All Fields] OR "recurrents"[All Fields]) AND ("drug implants"[Supplementary Concept] OR "drug implants"[All Fields] OR "implant"[All Fields] OR "drug implants"[MeSH Terms] OR ("drug"[All Fields] AND "implants"[All Fields]) OR "embryo implantation"[MeSH Terms] OR ("embryo"[All Fields] AND "implantation"[All Fields]) OR "embryo implantation"[All Fields] OR "implantation"[All Fields] OR "implant s"[All Fields] OR "implantability"[All Fields] OR "implantable"[All Fields] OR "implantables"[All Fields] OR "implantate"[All Fields] OR "implantated"[All Fields] OR "implantates"[All Fields] OR "implantations"[All Fields] OR "implanted"[All Fields] OR "implanter"[All Fields] OR "implanters"[All Fields] OR "implanting"[All Fields] OR "implantion"[All Fields] OR "implantitis"[All Fields] OR "implants"[All Fields]) AND ("failure"[All Fields] OR "failures"[All Fields])) OR ("rev infect"[Journal] OR "rif"[All Fields]) OR (("repeatabilities"[All Fields] OR "repeatability"[All Fields] OR "repeatable"[All Fields] OR "repeated"[All Fields] OR "repeatibility"[All Fields]) AND ("drug implants"[Supplementary Concept] OR "drug implants"[All Fields] OR "implant"[All Fields] OR "drug implants"[MeSH Terms] OR ("drug"[All Fields] AND "implants"[All Fields]) OR "embryo implantation"[MeSH Terms] OR ("embryo"[All Fields] AND "implantation"[All Fields]) OR "embryo implantation"[All Fields] OR "implantation"[All Fields] OR "implant s"[All Fields] OR "implantability"[All Fields] OR "implantable"[All Fields] OR "implantables"[All Fields] OR "implantate"[All Fields] OR "implantated"[All Fields] OR "implantates"[All Fields] OR "implantations"[All Fields] OR "implanted"[All Fields] OR "implanter"[All Fields] OR "implanters"[All Fields] OR "implanting"[All Fields] OR "implantion"[All Fields] OR "implantitis"[All Fields] OR "implants"[All Fields]) AND ("failure"[All Fields] OR "failures"[All Fields]))) AND (("preimplantation"[All Fields] OR "preimplantational"[All Fields] OR "preimplantion"[All Fields]) AND ("genetic testing"[MeSH Terms] OR ("genetic"[All Fields] AND "testing"[All Fields]) OR "genetic testing"[All Fields]))) OR ("Pre-implantation"[All Fields] AND ("genetic testing"[MeSH Terms] OR ("genetic"[All Fields] AND "testing"[All Fields]) OR "genetic testing"[All Fields]))) AND ("aneuploidy"[MeSH Terms] OR "aneuploidy"[All Fields] OR "aneuploidies"[All Fields])) AND ((excludepreprints[Filter] OR medline[Filter]) AND (humans[Filter]) AND (female[Filter]) AND (2016/4/1:2026/4/1[pdat]) AND (english[Filter])) | *147* |
| **#8** | **(((((Recurrent Implantation Failure) OR (RIF)) OR (Repeated Implantation Failure)) AND (Preimplantation Genetic Testing)) OR (Pre-implantation Genetic Testing)) AND (Aneuploidy) Filters: English, Humans, Female, Adult: 19-44 years, Adult: 19+ years, Young Adult: 19-24 years, Exclude preprints, MEDLINE, from 2016/4/1 - 2026/4/1**  (((((("recurrance"[All Fields] OR "recurrence"[MeSH Terms] OR "recurrence"[All Fields] OR "recurrences"[All Fields] OR "recurrencies"[All Fields] OR "recurrency"[All Fields] OR "recurrent"[All Fields] OR "recurrently"[All Fields] OR "recurrents"[All Fields]) AND ("drug implants"[Supplementary Concept] OR "drug implants"[All Fields] OR "implant"[All Fields] OR "drug implants"[MeSH Terms] OR ("drug"[All Fields] AND "implants"[All Fields]) OR "embryo implantation"[MeSH Terms] OR ("embryo"[All Fields] AND "implantation"[All Fields]) OR "embryo implantation"[All Fields] OR "implantation"[All Fields] OR "implant s"[All Fields] OR "implantability"[All Fields] OR "implantable"[All Fields] OR "implantables"[All Fields] OR "implantate"[All Fields] OR "implantated"[All Fields] OR "implantates"[All Fields] OR "implantations"[All Fields] OR "implanted"[All Fields] OR "implanter"[All Fields] OR "implanters"[All Fields] OR "implanting"[All Fields] OR "implantion"[All Fields] OR "implantitis"[All Fields] OR "implants"[All Fields]) AND ("failure"[All Fields] OR "failures"[All Fields])) OR ("rev infect"[Journal] OR "rif"[All Fields]) OR (("repeatabilities"[All Fields] OR "repeatability"[All Fields] OR "repeatable"[All Fields] OR "repeated"[All Fields] OR "repeatibility"[All Fields]) AND ("drug implants"[Supplementary Concept] OR "drug implants"[All Fields] OR "implant"[All Fields] OR "drug implants"[MeSH Terms] OR ("drug"[All Fields] AND "implants"[All Fields]) OR "embryo implantation"[MeSH Terms] OR ("embryo"[All Fields] AND "implantation"[All Fields]) OR "embryo implantation"[All Fields] OR "implantation"[All Fields] OR "implant s"[All Fields] OR "implantability"[All Fields] OR "implantable"[All Fields] OR "implantables"[All Fields] OR "implantate"[All Fields] OR "implantated"[All Fields] OR "implantates"[All Fields] OR "implantations"[All Fields] OR "implanted"[All Fields] OR "implanter"[All Fields] OR "implanters"[All Fields] OR "implanting"[All Fields] OR "implantion"[All Fields] OR "implantitis"[All Fields] OR "implants"[All Fields]) AND ("failure"[All Fields] OR "failures"[All Fields]))) AND (("preimplantation"[All Fields] OR "preimplantational"[All Fields] OR "preimplantion"[All Fields]) AND ("genetic testing"[MeSH Terms] OR ("genetic"[All Fields] AND "testing"[All Fields]) OR "genetic testing"[All Fields]))) OR ("Pre-implantation"[All Fields] AND ("genetic testing"[MeSH Terms] OR ("genetic"[All Fields] AND "testing"[All Fields]) OR "genetic testing"[All Fields]))) AND ("aneuploidy"[MeSH Terms] OR "aneuploidy"[All Fields] OR "aneuploidies"[All Fields])) AND ((excludepreprints[Filter] OR medline[Filter]) AND (humans[Filter]) AND (female[Filter]) AND (2016/4/1:2026/4/1[pdat]) AND (english[Filter]) AND (adult[Filter] OR alladult[Filter] OR youngadult[Filter])) | *69* |

***1.2. Filters applied for fields:***

PUBLICATION DATE – 2016/04/01 to 2026/04/01

ARTICLE LANGUAGE – English

SPECIES – Humans

SEX – Female

AGE – Adult: 19+ years, Young Adult: 19-24 years, Adult: 19-44 years

OTHER: Exclude preprints, MEDLINE

**2. Web of Science^TM^ (WOS) (Clarivate Analytics, 1997)**

***2.1. Extensive searching string***

| **#1** | (((((ALL=(Recurrent Implantation Failure)) OR ALL=(RIF)) OR ALL=(Repeated Implantation Failure)) AND ALL=(Preimplantation Genetic Testing)) OR ALL=(Pre-implantation Genetic Testing)) AND ALL=(Aneuploidy) | *251* |
| --- | --- | --- |
| **#2** | (((((ALL=(Recurrent Implantation Failure)) OR ALL=(RIF)) OR ALL=(Repeated Implantation Failure)) AND ALL=(Preimplantation Genetic Testing)) OR ALL=(Pre-implantation Genetic Testing)) AND ALL=(Aneuploidy) and 2016 or 2017 or 2018 or 2019 or 2020 or 2021 or 2022 or 2023 or 2024 or 2025 or 2026 (Publication Years) | *219* |
| **#3** | (((((ALL=(Recurrent Implantation Failure)) OR ALL=(RIF)) OR ALL=(Repeated Implantation Failure)) AND ALL=(Preimplantation Genetic Testing)) OR ALL=(Pre-implantation Genetic Testing)) AND ALL=(Aneuploidy) and 2016 or 2017 or 2018 or 2019 or 2020 or 2021 or 2022 or 2023 or 2024 or 2025 or 2026 (Publication Years) and Article (Document Types) | *145* |
| **#4** | (((((ALL=(Recurrent Implantation Failure)) OR ALL=(RIF)) OR ALL=(Repeated Implantation Failure)) AND ALL=(Preimplantation Genetic Testing)) OR ALL=(Pre-implantation Genetic Testing)) AND ALL=(Aneuploidy) and 2016 or 2017 or 2018 or 2019 or 2020 or 2021 or 2022 or 2023 or 2024 or 2025 or 2026 (Publication Years) and Article (Document Types) and Science Citation Index Expanded (SCI-EXPANDED) or Emerging Sources Citation Index (ESCI) (Web of Science Index) | *145* |
| **#5** | (((((ALL=(Recurrent Implantation Failure)) OR ALL=(RIF)) OR ALL=(Repeated Implantation Failure)) AND ALL=(Preimplantation Genetic Testing)) OR ALL=(Pre-implantation Genetic Testing)) AND ALL=(Aneuploidy) and 2016 or 2017 or 2018 or 2019 or 2020 or 2021 or 2022 or 2023 or 2024 or 2025 or 2026 (Publication Years) and Article (Document Types) and Science Citation Index Expanded (SCI-EXPANDED) or Emerging Sources Citation Index (ESCI) (Web of Science Index) and English (Languages) | *145* |

***2.2. Filters applied for fields:***

PUBLICATION YEARS – 2014-2026

DOCUMENT TYPES – Article

LANGUAGE – English

WEB OF SCIENCE INDEX – Science Citation Index Expanded (SCI-EXPANDED), Emerging Sources Citation Index (ESCI)

**3. Scopus (Elsevier, 2004)**

***3.1. Extensive searching string:***

| **#1** | TITLE-ABS-KEY ( Recurrent Implantation Failure OR RIF OR Repeated Implantation Failure ) AND TITLE-ABS-KEY ( Preimplantation Genetic Testing OR Pre-implantation Genetic Testing ) AND TITLE-ABS-KEY ( Aneuploidy ) | *111* |
| --- | --- | --- |
| **#2** | TITLE-ABS-KEY ( Recurrent Implantation Failure OR RIF OR Repeated Implantation Failure ) AND TITLE-ABS-KEY ( Preimplantation Genetic Testing OR Pre-implantation Genetic Testing ) AND TITLE-ABS-KEY ( Aneuploidy ) AND PUBYEAR > 2015 AND PUBYEAR < 2027 | *99* |
| **#3** | TITLE-ABS-KEY ( Recurrent Implantation Failure OR RIF OR Repeated Implantation Failure ) AND TITLE-ABS-KEY ( Preimplantation Genetic Testing OR Pre-implantation Genetic Testing ) AND TITLE-ABS-KEY ( Aneuploidy ) AND PUBYEAR > 2015 AND PUBYEAR < 2027 AND ( LIMIT-TO ( DOCTYPE , "ar" ) ) | *70* |
| **#4** | TITLE-ABS-KEY ( Recurrent Implantation Failure OR RIF OR Repeated Implantation Failure ) AND TITLE-ABS-KEY ( Preimplantation Genetic Testing OR Pre-implantation Genetic Testing ) AND TITLE-ABS-KEY ( Aneuploidy ) AND PUBYEAR > 2015 AND PUBYEAR < 2027 AND ( LIMIT-TO ( DOCTYPE , "ar" ) ) AND ( LIMIT-TO ( PUBSTAGE , "final" ) ) | *70* |
| **#5** | TITLE-ABS-KEY ( Recurrent Implantation Failure OR RIF OR Repeated Implantation Failure ) AND TITLE-ABS-KEY ( Preimplantation Genetic Testing OR Pre-implantation Genetic Testing ) AND TITLE-ABS-KEY ( Aneuploidy ) AND PUBYEAR > 2015 AND PUBYEAR < 2027 AND ( LIMIT-TO ( DOCTYPE , "ar" ) ) AND ( LIMIT-TO ( PUBSTAGE , "final" ) ) AND ( LIMIT-TO ( SRCTYPE , "j" ) ) | *70* |
| **#6** | TITLE-ABS-KEY ( Recurrent Implantation Failure OR RIF OR Repeated Implantation Failure ) AND TITLE-ABS-KEY ( Preimplantation Genetic Testing OR Pre-implantation Genetic Testing ) AND TITLE-ABS-KEY ( Aneuploidy ) AND PUBYEAR > 2015 AND PUBYEAR < 2027 AND ( LIMIT-TO ( DOCTYPE , "ar" ) ) AND ( LIMIT-TO ( PUBSTAGE , "final" ) ) AND ( LIMIT-TO ( SRCTYPE , "j" ) ) AND ( LIMIT-TO ( LANGUAGE , "English" ) ) | *66* |

***3.2. Filters applied for fields:***

YEAR – 2016-2026

DOCUMENT TYPE – Article

PUBLICATION STAGE – Final

SOURCE TYPE – Journal

LANGUAGE – English

**4. Excerpta Medica dataBASE (EMBASE) (Elsevier, 1947)**

***4.1. Extensive searching string:***

| **#1** | ((recurrent AND ('implantation'/exp OR implantation) AND ('failure'/exp OR failure) OR rif OR repeated) AND ('implantation'/exp OR implantation) AND ('failure'/exp OR failure) AND preimplantation AND ('genetic'/exp OR genetic) AND ('testing'/exp OR testing) OR 'pre implantation') AND ('genetic'/exp OR genetic) AND ('testing'/exp OR testing) AND ('aneuploidy'/exp OR aneuploidy) | *594* |
| --- | --- | --- |
| **#2** | ((recurrent AND ('implantation' OR 'implantation'/exp OR implantation) AND ('failure' OR 'failure'/exp OR failure) OR rif OR repeated) AND ('implantation' OR 'implantation'/exp OR implantation) AND ('failure' OR 'failure'/exp OR failure) AND preimplantation AND ('genetic' OR 'genetic'/exp OR genetic) AND ('testing' OR 'testing'/exp OR testing) OR 'pre implantation') AND ('genetic' OR 'genetic'/exp OR genetic) AND ('testing' OR 'testing'/exp OR testing) AND ('aneuploidy' OR 'aneuploidy'/exp OR aneuploidy) AND [2016-2026]/py | *550* |
| **#3** | ((recurrent AND ('implantation' OR 'implantation'/exp OR implantation) AND ('failure' OR 'failure'/exp OR failure) OR rif OR repeated) AND ('implantation' OR 'implantation'/exp OR implantation) AND ('failure' OR 'failure'/exp OR failure) AND preimplantation AND ('genetic' OR 'genetic'/exp OR genetic) AND ('testing' OR 'testing'/exp OR testing) OR 'pre implantation') AND ('genetic' OR 'genetic'/exp OR genetic) AND ('testing' OR 'testing'/exp OR testing) AND ('aneuploidy' OR 'aneuploidy'/exp OR aneuploidy) AND ([embase]/lim OR [medline]/lim OR 'clinical trial':dtype OR [pubmed-not-medline]/lim) AND [2016-2026]/py | *547* |
| **#4** | ((recurrent AND ('implantation' OR 'implantation'/exp OR implantation) AND ('failure' OR 'failure'/exp OR failure) OR rif OR repeated) AND ('implantation' OR 'implantation'/exp OR implantation) AND ('failure' OR 'failure'/exp OR failure) AND preimplantation AND ('genetic' OR 'genetic'/exp OR genetic) AND ('testing' OR 'testing'/exp OR testing) OR 'pre implantation') AND ('genetic' OR 'genetic'/exp OR genetic) AND ('testing' OR 'testing'/exp OR testing) AND ('aneuploidy' OR 'aneuploidy'/exp OR aneuploidy) AND [humans]/lim AND [english]/lim AND [clinical study]/lim AND ([embase]/lim OR [medline]/lim OR 'clinical trial':dtype OR [pubmed-not-medline]/lim) AND [2016-2026]/py | *428* |
| **#5** | ((recurrent AND ('implantation' OR 'implantation'/exp OR implantation) AND ('failure' OR 'failure'/exp OR failure) OR rif OR repeated) AND ('implantation' OR 'implantation'/exp OR implantation) AND ('failure' OR 'failure'/exp OR failure) AND preimplantation AND ('genetic' OR 'genetic'/exp OR genetic) AND ('testing' OR 'testing'/exp OR testing) OR 'pre implantation') AND ('genetic' OR 'genetic'/exp OR genetic) AND ('testing' OR 'testing'/exp OR testing) AND ('aneuploidy' OR 'aneuploidy'/exp OR aneuploidy) AND ('article'/it OR 'clinical trial'/it) AND [humans]/lim AND [english]/lim AND [clinical study]/lim AND ([embase]/lim OR [medline]/lim OR 'clinical trial':dtype OR [pubmed-not-medline]/lim) AND [2016-2026]/py | *127* |
| **#6** | ((recurrent AND ('implantation' OR 'implantation'/exp OR implantation) AND ('failure' OR 'failure'/exp OR failure) OR rif OR repeated) AND ('implantation' OR 'implantation'/exp OR implantation) AND ('failure' OR 'failure'/exp OR failure) AND preimplantation AND ('genetic' OR 'genetic'/exp OR genetic) AND ('testing' OR 'testing'/exp OR testing) OR 'pre implantation') AND ('genetic' OR 'genetic'/exp OR genetic) AND ('testing' OR 'testing'/exp OR testing) AND ('aneuploidy' OR 'aneuploidy'/exp OR aneuploidy) AND ('article'/it OR 'clinical trial'/it) AND [female]/lim AND [humans]/lim AND [english]/lim AND [clinical study]/lim AND ([embase]/lim OR [medline]/lim OR 'clinical trial':dtype OR [pubmed-not-medline]/lim) AND [2016-2026]/py | *118* |
| **#7** | ((recurrent AND ('implantation' OR 'implantation'/exp OR implantation) AND ('failure' OR 'failure'/exp OR failure) OR rif OR repeated) AND ('implantation' OR 'implantation'/exp OR implantation) AND ('failure' OR 'failure'/exp OR failure) AND preimplantation AND ('genetic' OR 'genetic'/exp OR genetic) AND ('testing' OR 'testing'/exp OR testing) OR 'pre implantation') AND ('genetic' OR 'genetic'/exp OR genetic) AND ('testing' OR 'testing'/exp OR testing) AND ('aneuploidy' OR 'aneuploidy'/exp OR aneuploidy) AND ('article'/it OR 'clinical trial'/it) AND [female]/lim AND ([adult]/lim OR [young adult]/lim) AND [humans]/lim AND [english]/lim AND [clinical study]/lim AND ([embase]/lim OR [medline]/lim OR 'clinical trial':dtype OR [pubmed-not-medline]/lim) AND [2016-2026]/py | *99* |

***4.2. Filters applied for fields:***

MAPPING – Map to preferred term in Emtree, Search also as free text in all fields, Explode using narrower Emtree terms, Search as broadly as possible

DATE – 01/04/2016 to 01/04/2026

SOURCES – Embase, MEDLINE, Clinical Trials, PubMed-not-MEDLINE

QUICK LIMITS – Humans, Clinical studies, Only in English

PUBLICATION TYPES – Article, Clinical Trial

GENDER – Female

AGE – Adult (18-64 years), Young adult (18-24 years)
